# A comprehensive and systematic analysis of Dihydrolipoamide S-acetyltransferase *(DLAT)* as a novel prognostic biomarker in pan-cancer and glioma

**DOI:** 10.32604/or.2024.048138

**Published:** 2024-11-13

**Authors:** HUI ZHOU, ZHENGYU YU, JING XU, ZHONGWANG WANG, YALI TAO, JINJIN WANG, PEIPEI YANG, JINRONG YANG, TING NIU

**Affiliations:** Department of Hematology, West China Hospital, Sichuan University, Chengdu, 610041, China

**Keywords:** Dihydrolipoamide S-acetyltransferase (DLAT), Glioma, Prognostic, Immunological

## Abstract

**Background:**

Dihydrolipoamide S-acetyltransferase (*DLAT*) is a subunit of the pyruvate dehydrogenase complex (PDC), a rate-limiting enzyme complex, that can participate in either glycolysis or the tricarboxylic acid cycle (TCA). However, the pathogenesis is not fully understood. We aimed to perform a more systematic and comprehensive analysis of *DLAT* in the occurrence and progression of tumors, and to investigate its function in patients’ prognosis and immunotherapy.

**Methods:**

The differential expression, diagnosis, prognosis, genetic and epigenetic alterations, tumor microenvironment, stemness, immune infiltration cells, function enrichment, single-cell analysis, and drug response across cancers were conducted based on multiple computational tools. Additionally, we validated its carcinogenic effect and possible mechanism in glioma cells.

**Results:**

We exhibited that *DLAT* expression was increased in most tumors, especially in glioma, and affected the survival of tumor patients. *DLAT* was related to RNA modification genes, DNA methylation, immune infiltration, and immune infiltration cells, including CD4+ T cells, CD8+ T cells, Tregs, and cancer-associated fibroblasts. Single-cell analysis displayed that *DLAT* might regulate cancer by mediating angiogenesis, inflammation, and stemness. Enrichment analysis revealed that *DLAT* might take part in the cell cycle pathway. Increased expression of *DLAT* leads tumor cells to be more resistant to many kinds of compounds, including PI3Kβ inhibitors, PKC inhibitors, HSP90 inhibitors, and MEK inhibitors. In addition, glioma cells with *DLAT* silence inhibited proliferation, migration, and invasion ability, and promoted cell apoptosis.

**Conclusion:**

We conducted a comprehensive analysis of *DLAT* in the occurrence and progression of tumors, and its possible functions and mechanisms. *DLAT* is a potential diagnostic, prognostic, and immunotherapeutic biomarker for cancer patients.

## Introduction

Nowadays, the malignant tumor has become the primary cause of human death, and seriously affect the quality of life of patients. In addition to surgery, chemotherapy, radiotherapy, and targeted therapy, immunotherapy is also one of the crucial treatments for cancer patients [1]. However, the objective response rates are unsatisfactory in many cancer patients, and some patients often resist or relapse [2,3]. Therefore, it is necessary to explore novel targets and investigate their correlations with patients’ prognosis and tumor immunity.

Dihydrolipoamide S-acetyltransferase (*DLAT*) is subunit E2 of the pyruvate dehydrogenase complex (PDC) [4], which is a mitochondrial multienzyme complex that can participate in either glycolysis or the tricarboxylic acid cycle (TCA) cycle [5,6]. The reprogramming of cell metabolism is usually observed in cancer cells [7,8]. Cancer cells absorb and utilize much more glucose than normal cells, and take advantage of glycolysis metabolism than oxidative phosphorylation regardless of oxygen availability, this phenomenon was known as the Warburg effect or aerobic glycolysis [9]. A previous study reported that *DLAT* expression was increased in gastric cancer cells [6]. Besides, Chen et al. discovered that *DLAT* was overexpressed in non-small cell lung cancer (NSCLC), and inhibited acetyl-CoA production but promoted L-lactate and pyruvate production [10]. These results strongly demonstrated that *DLAT* contributed to tumorigenesis by promoting glycolysis metabolism. Additionally, *DLAT* is significantly elevated in osteosarcoma cell lines compared with normal osteoblast cell lines [11]. While, several bioinformatics studies proved that *DLAT* was expressed at low levels in clear cell renal cell carcinoma [12]. Furthermore, *DLAT* participated in the development and prognosis of breast invasive carcinoma (BRCA) [13] and colon adenocarcinoma (COAD) [14]. Nevertheless, specific studies on *DLAT* in tumors are few and lack systematic pan-cancer investigation. Consequently, exploring the role of *DLAT* expression and alterations in cancers was extremely urgent.

Therefore, a systematic and comprehensive analysis to evaluate the expression, gene and epigenetic alteration, methylation, and clinical, and prognostic profiles of *DLAT* across cancers. Moreover, we analyzed its relationship with immune cells, immune genes, tumor microenvironment, and tumor stemness score. In addition, enrichment analysis, single-cell analysis, and drug responses related to *DLAT* were analyzed. Moreover, we validated the function of *DLAT* in glioma cells. In conclusion, our results identified the role of *DLAT* across cancers and suggested that *DLAT* was a prognostic and immunotherapeutic biomarker in cancer patients.

## Materials and Methods

### Data acquisition

We downloaded RNA and clinical data from The Cancer Genome Atlas (TCGA), TARGET, and Genotype-Tissue Expression (GTEx) from the UCSC database. In addition, we acquired prognostic data for TCGA from a previous study [15]. Meanwhile, we also obtained the TARGET follow-up data as a supplement from the UCSC. We also downloaded the RNA-seq data of the 325 glioma samples from the Chinese Glioma Genome Atlas (CGGA) database (http://www.cgga.org.cn/). The tumor cell line RNA expression data was downloaded from The Cancer Cell Line Encyclopedia (CCLE). The protein expression of DLAT was evaluated by the Human Protein Atlas (HPA). Suppl. Table S1 lists abbreviations of tumors.

### Clinical characteristics analysis

We developed the Cox proportional hazards regression model to analyze overall survival (OS), disease-specific survival (DSS), disease-free interval (DFI), and progression-free interval (PFI) of *DLAT* across cancers. Kaplan‒Meier analysis was performed to analyze the patient’s prognosis. The CGGA dataset was also used to analyze the survival of *DLAT* in glioma patients.

The diagnostic significance of *DLAT* across cancers was assessed by the Receiver Operator Characteristic (ROC) curve via “pROC” (v1.17.0.1). The diagnosis accuracy was evaluated by the Area under Curve (AUC). The AUC is closer to 1, the diagnosis accuracy is better. The clinical value of *DLAT* was calculated by unpaired Wilcoxon rank sum, signed rank, and Kruskal‒Wallis tests.

The associations between *DLAT* expression and molecular or immune subtypes across cancers were analyzed by the TISIDB database. There are six immune subtypes, C1 meaning wound healing subtype, C2 representing the IFN-γ dominant subtype, C3 meaning inflammatory subtype, C4 representing lymphocyte depletion, C5 meaning immunologically quiet, and C6 representing the TGF-β dominant subtype.

The IC_50_ values of various compounds in cancer cell lines were obtained from the GDSC dataset (https://www.cancerrxgene.org), to assess the relationship between *DLAT* and the drug response of tumor cells by the Spearman correlation coefficient.

### Genetic and epigenetic alterations

The genomic alteration analyses were used by the cBioPortal database (https://www.cbioportal.org/). The mRNA methylation was an important posttranscriptional gene regulation in eukaryotes. The RNA methylation modifications included methylation of N6 adenosine (m6A), N1 methyladenosine (m1A), and 5-methylcytosine (m5C), participating in cell differentiation, development, and progression, and so on [16]. The relationships between forty-four marker genes of RNA modification, including m1A, m5C, and m6A, and *DLAT* expression were evaluated.

Mismatch repair (MMR) genes downregulated or functionally defective can cause irreparable DNA replication mistakes and somatic mutations, therefore increasing the incidence rate of cancer [17]. A correlation analysis was conducted.

The correlation between *DLAT* expression and methylation was evaluated via the cBioPortal database. Furthermore, the relationship between *DLAT* methylation and patients’ prognosis was also assessed. Additionally, the expression of *DLAT* promotor methylation between cancers and normal tissues was investigated.

### Tumor microenvironment analysis

We downloaded all level 4 Simple Nucleotide Variation data of TCGA samples from GDC (https://portal.gdc.cancer.gov/). Tumor mutation burden (TMB) can reflect the proportion of somatic mutations in tumors and is a quantitative biological marker of the immune response [18]. We calculated the TMB by the R MAftools package. Microsatellite instability (MSI) is the arbitrary length change of microsatellites in cancer tissue due to the insertion or deletion of repeat units compared with normal tissue, and it is a very important molecular biomarker in almost all solid tumors [19]. The tumor purity was acquired from the previous study [20]. The tumor stemness score was obtained by calculating the methylation-based DNA stemness score (DNAss) and expression-based RNA stemness score (RNAss) index of methylation characteristics in diverse tumors [21].

### Tumor immune microenvironment analysis

We obtained 10,180 tumor samples from 44 cancer types for immune infiltration analysis. Estimation of Stromal and Immune Cells in Malignant Tumor Tissues Using Expression Data (ESTIMATE) was used to reflect the level of stromal or immune cell infiltrations. The analysis used the R software packages “estimate” and “psych”.

The correlation between *DLAT* expression and immune infiltrating cells in tumors was performed by the TIMER2 tool (http://timer.cistrome.org/). CD4+ T cells, CD8+ T cells, Tregs, and cancer-associated fibroblasts were chosen for detailed analysis by the TIMER, CIBERSORT, CIBERSORT-ABS, QUANTISEQ, XCELL, MCPCOUNTER, and EPIC algorithms. We used the ssGSEA algorithm to evaluate 31 infiltrating cells in glioma as previously described [22].

Besides, the association between *DLAT* expression and immune-related genes in five immune pathways was evaluated.

### Single-cell and enrichment analysis

We analyzed the function of *DLAT* at the single-cell level by CancerSEA [23]. The correlation was >0.3 and the *p*-value was <0.05. We compared the single-cell expression and distribution of *DLAT* among patients with high-grade gliomas (HGGs) utilizing the TISCH database.

The top 50 *DLAT*-binding proteins were downloaded from the STRING database. GEPIA2 was used to acquire the top 100 *DLAT*-related target genes. An intersection analysis was evaluated by the Venn plot. Gene Ontology (GO) and KEGG pathway enrichment analyses were used to examine the biological and molecular functions of the two sets of data. GSEA analysis was used to investigate the potential function of *DLAT* in cancers.

### Gene silencing

The GBM cell line A172 came from the American Type Culture Collection (ATCC) and was cultured in DMEM (Gibco, Grand Island, NY, USA) with 10% fetal calf serum (Gibco) and 1.0 mmol/L penicillin–streptomycin combination (Hyclone). By utilizing the INTERFERin^®^ reagent from Poly-Plus Corporation, we transfected siRNAs and negative control into A172 cells. After a 48-h incubation period, the cells were collected and prepared for subsequent experiments. The sequences for *DLAT* siRNA were: 5′-AAGTTCTTCTTGTCTTTCCAGATAT-3′ and 5′-TATAGTGGAAAGAGAAGGAGTAAG-3′ (Tsingke Biotech). All cells were appraised by STR and performed mycoplasma testing.

### Extraction of total RNA and qRT-PCR

The total RNA was extracted using the Trizol reagent (Ambion, Austin, Texas, USA). The extracted RNA was converted into cDNA following the instructions (Genecopoeia, Rockville, MD, USA). The qRT-PCR reactions were performed using the BlazeTaqTM SYBR^®^ Green qPCR Mix 2.0 kit (Genecopoeia) and the corresponding reaction system. The forward primer sequence was GTGTTGCGGTCAGTACTCCT, and the reverse primer sequence was CGTAAAAGTGCCACCCTGGA (Tsingke Biotech, Beijing, China).

### Western blotting

To isolate the proteins, the GBM cell lines were lysed by RIPA buffer (Beyotime, Shanghai, China) with a cocktail (Thermo Scientific, Waltham, MA, USA) added. The protein was collected by centrifuge and the quantity was accurately measured using a BCA assay (Abcam). The antibodies included anti-DLAT (Cell Signaling Technology, 1:1000 dilution) and anti-ACTIN (Abcam, 1:1000 dilution). Cellular proteins were extracted using a lysis buffer, incubated on ice for 30 min with intermittent vortexing, and clarified by centrifugation at 14,000 × g for 15 min at 4°C. Protein concentrations were determined using the bicinchoninic acid (BCA) assay kit. Equal amounts of protein (20 μg per sample) were denatured by boiling with loading buffer at 95°C for 5 min, separated by SDS-PAGE on a 12% polyacrylamide gel at 120 V for approximately 1 h, and transferred onto PVDF membranes using a semi-dry transfer system at 15 V for 45 minutes. Membranes were blocked with 5% non-fat dry milk in TBS-T for 1 h at room temperature, then incubated overnight at 4°C with primary antibodies specific to the target proteins, diluted in blocking buffer. After washing with TBS-T, membranes were incubated with HRP-conjugated secondary antibodies for 1 h at room temperature. Immunoreactive bands were visualized using an enhanced chemiluminescence detection reagent on a chemiluminescent imaging system.

### Proliferation analysis

2.0 × 10^4^ cells were transfected and cultured in a 96-well plate. Following 48 h of growth and cultivation, a WST-8 solution (the Enhanced Cell Counting Kit-8, diluted 1:10) was introduced and incubated for 2 h. The Optical Density (OD) value was then accurately determined using a sophisticated microplate reader.

### Apoptosis analysis

Cells underwent a 48-h transfection with siRNA, then cells were collected by centrifuge, washed with PBS, and treated with Annexin V/FITC and Propidium Iodide (BD Biosciences, San Jose, CA, USA), and then detected by flow cytometry (BD Biosciences) and analyzed by FlowJo.

### Transwell assay

3 × 10^5^ and 5 × 10^5^ cells in serum-free medium were added on Transwell membranes (5 μm pore size, Costar). The medium with 10% FBS was added to the lower chambers. The membranes were bedded with Matrigel (BD Biosciences) in advance for the invasion analysis. After 24 h, cells on the upper membranes were fixed by 4% paraformaldehyde (Thermo Scientific) and stained with crystal violet (Beyotime). The cells on the membrane were observed by microscope (Nikon). A flow cytometer (BD Biosciences) was used to quantify the number of cells in the lower chambers.

### Statistical analysis

All analyses were performed by R software (version 4.2.1). The Wilcoxon’s test and analysis of variance (ANOVA) were used for the two groups and multiple groups, respectively. The correlation analysis was calculated by Spearman’s correlation test. *p < 0.05; **p < 0.01; ***p < 0.001; ****p < 0.0001; and ns, not significant.

## Results

### Alterations of DLAT in pan-cancer

*DLAT* physiologically exhibited the highest expression level in heart muscle and skeletal muscle but exhibited low expression levels in most other normal tissues (Suppl. Fig. S1A). Suppl. Fig. S1B showed that the *DLAT* expression was highest in the lymphoid U-698 cell line and generally higher in some lymphoid, myeloid, and female reproductive system cell lines. Moreover, *DLAT* expression was lowest in liver cancer and was greatest in colorectal cancer (Suppl. Fig. S1C).

**Figure 1:**
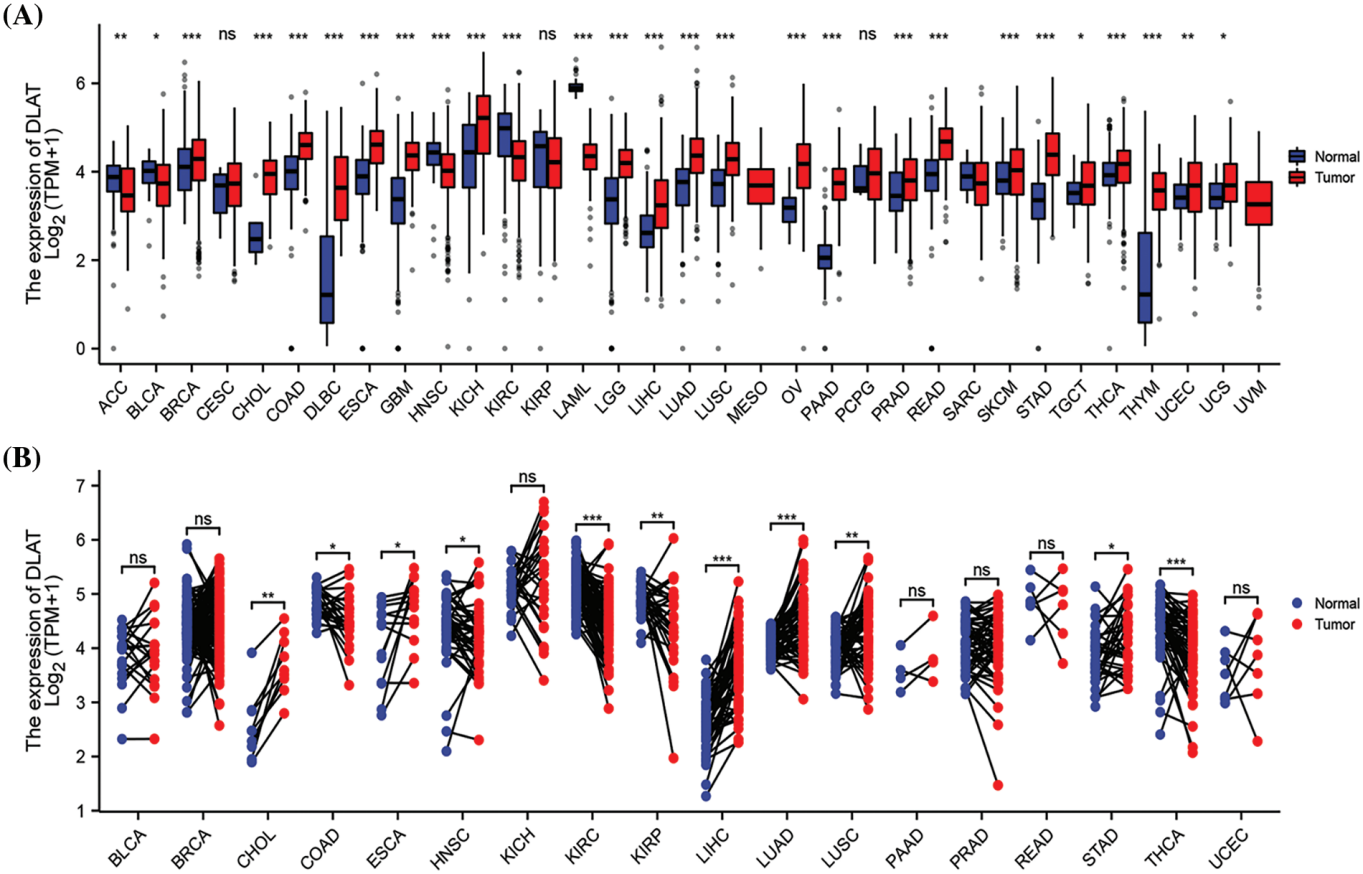
Differential analysis of *DLAT* expression across cancers. (A) *DLAT* expression between tumor and normal samples from the GTEx and TCGA databases. (B) *DLAT* expression in matched tumor and normal samples from XENA and TCGA. ns: not significant; *p < 0.05; **p < 0.01; ***p < 0.001.

We evaluated *DLAT* gene expression in 34 cancer species in TCGA, TARGET, and GTEx pan-cancer. The *DLAT* gene was highly expressed in 22 types of cancers, including glioblastoma multiforme (GBM), lower grade glioma (LGG), and kidney chromophobe (KICH). In comparison, *DLAT* was lowly expressed in 5 types of cancers: adrenocortical carcinoma (ACC), bladder urothelial carcinoma (BLCA), head and Neck squamous cell carcinoma (HNSC), kidney renal clear cell carcinoma (KIRC), and acute myeloid leukemia (LAML) (Fig. 1A). For paired tumors and adjacent normal tissues in TGCA, *DLAT* was lowly expressed in COAD, HNSC, KIRC, Kidney renal papillary cell carcinoma (KIRP), and thyroid carcinoma (THCA) and highly expressed in six types of cancer (Fig. 1B).

Genetic and epigenetic alterations might influence the expression and have been closely associated with tumorigenesis. A high frequency of gene alterations was found in most patients except with ACC, cholangiocarcinoma (CHOL), Diffuse Large B-cell Lymphoma (DLBC), liver hepatocellular carcinoma (LIHC), mesothelioma (MESO), thymoma (THYM) and THCA. In addition, *DLAT* mutation frequencies were found to be the highest in uterine *corpus* endometrial carcinoma (UCEC), BLCA, colon adenocarcinoma/rectum adenocarcinoma esophageal carcinoma (COADREAD), skin cutaneous melanoma (SKCM), and stomach adenocarcinoma (STAD) (Suppl. Fig. S2A). The main type of genetic alteration was the missense mutation of *DLAT* (Suppl. Fig. S2B). The 3D structure of the *DLAT* protein is shown in Suppl. Fig. S2C.

**Figure 2:**
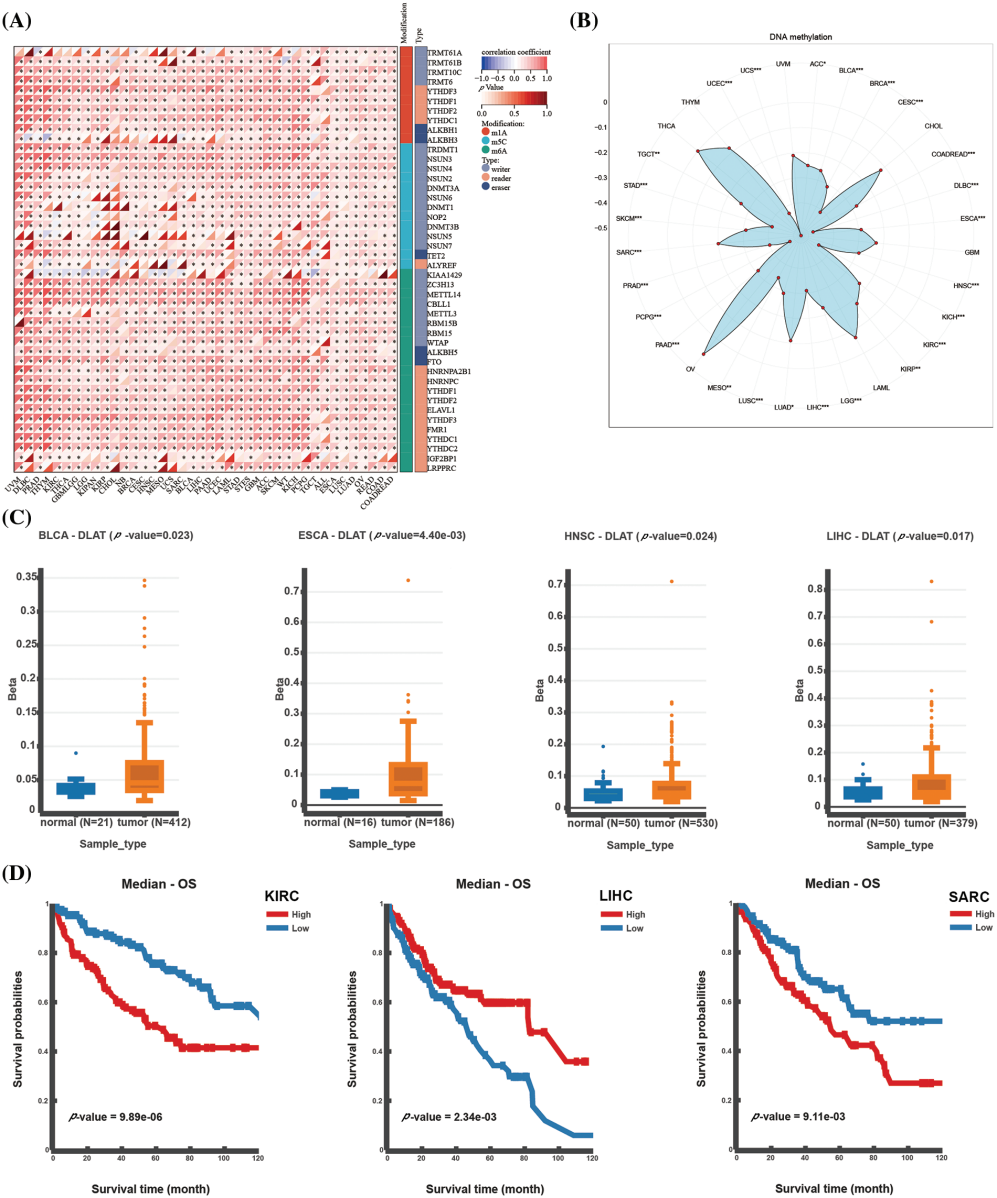
Epigenetic alterations of *DLAT*. (A) Correlation between *DLAT* and RNA-modified genes. (B) Radar plot showing the correlation between *DLAT* and promotor methylation. (C) Differential expression of *DLAT* promoter methylation levels across cancers. (D) Kaplan‒Meier curves exhibiting the correlations of *DLAT* promoter methylation levels and OS. *p < 0.05; **p < 0.01; ***p < 0.001.

RNA modification is a common intracellular chemical modification, and it participates in various pathological processes, such as immune system diseases and cancer. *DLAT* expression was significantly positively correlated with RNA modification genes in almost all tumors (Fig. 2A). DNA methylation is also one of the common epigenetic regulators. We demonstrated significant negative relationships between *DLAT* expression and methylation in most tumors (Fig. 2B). Moreover, we evaluated the differential expression of *DLAT* promoter methylation levels between cancers and normal tissue. The results exhibited a high methylation level of *DLAT* in BLCA, esophageal carcinoma (ESCA), HNSC, and LIHC tissues compared to normal tissues (Fig. 2C). Furthermore, Increased *DLAT* methylation was related to shorter OS in patients with KIRC and sarcoma (SARC), while was correlated with longer OS in patients with LIHC (Fig. 2D). These results identified that *DLAT* might influence tumor development by regulating the repair of RNA and DNA methylation across cancers.

### Clinical characteristics of DLAT in pan-cancer

**Figure 3:**
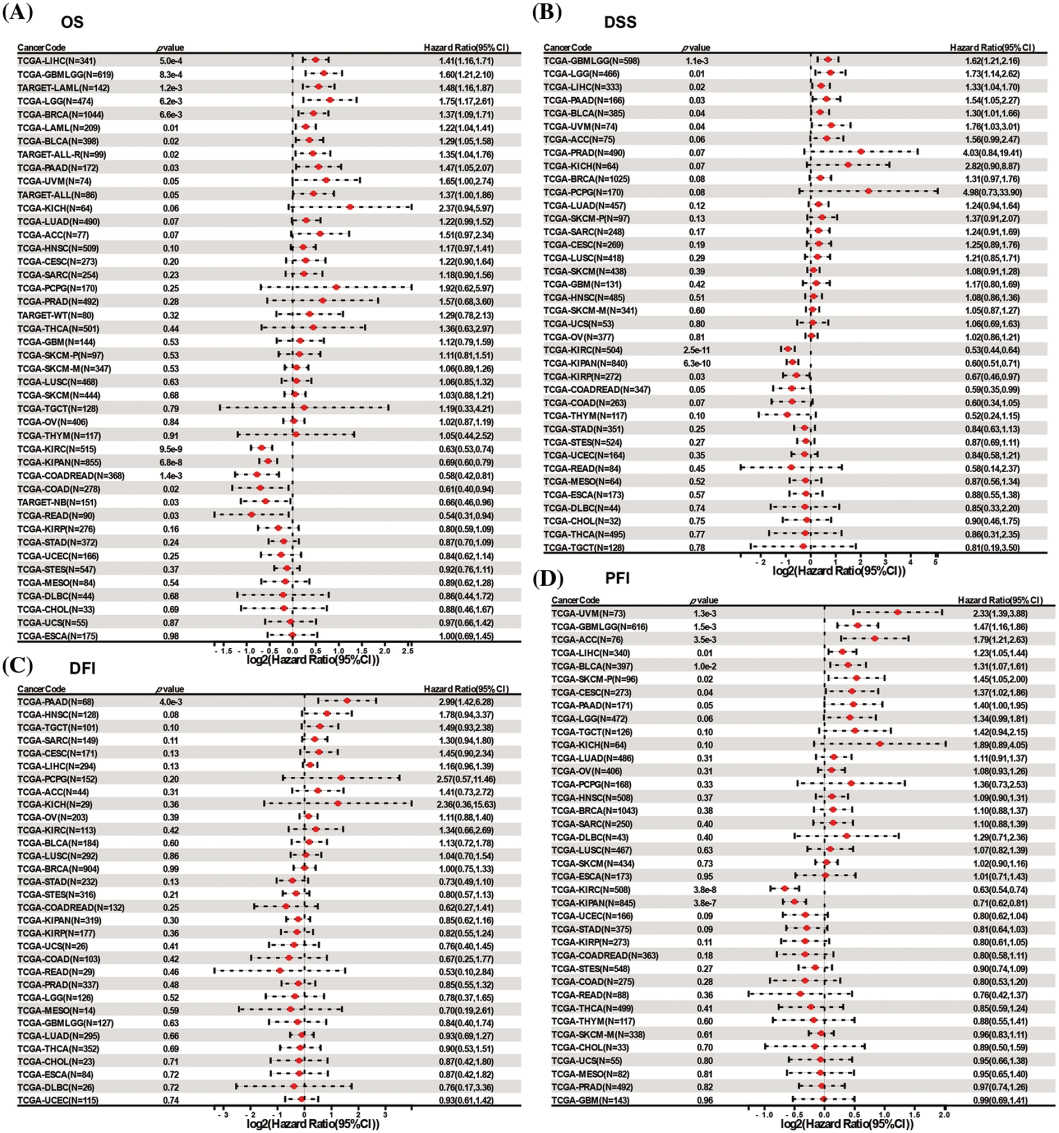
Forest plots of *DLAT* by univariate Cox regression analysis across cancers. (A) OS. (B) DSS. (C) DFI. (D) PFI.

To further assess the prognostic value of *DLAT* expression across cancers, we performed a Cox proportional hazards model analysis, including OS, DSS, DFI, and PFI. Univariate Cox regression analysis of OS identified that *DLAT* was a risk factor for patients with LIHC, glioma (GBMLGG), TARGET-LAML, LGG, BRCA, LAML, BLCA, recurrence acute lymphoblastic leukemia (TARGET-ALL-R) and pancreatic adenocarcinoma (PAAD) and benefit for patients with KIRC, Pan-kidney cohort (KICH+KIRC+KIRP) (KIPAN), COADREAD, COAD, neuroblastoma (TARGET-NB) and rectum adenocarcinoma (READ) (Fig. 3A). The DSS analysis demonstrated that *DLAT* was related to poor survival in patients with GBMLGG, LGG, LIHC, PAAD, BLCA, and uveal melanoma (UVM) and was correlated with favorable survival in patients with KIRC, KIPAN, and KIRP (Fig. 3B). The DFI analysis showed that *DLAT* was an adverse prognostic factor for patients with PAAD (Fig. 3C). The PFI analysis demonstrated that *DLAT* was a unfavorable factor for patients with UVM, GBMLGG, ACC, LIHC, BLCA, SKCM-P, and cervical squamous cell carcinoma and endocervical adenocarcinoma (CESC) and benefit for patients with KIRC and KIPAN (Fig. 3D).

Furthermore, Kaplan‒Meier survival analyses were explored across cancers. The OS analysis showed that elevated *DLAT* expression levels were an unfavorable factor in ACC, BRCA, GBMLGG, LGG, LIHC, PAAD, SKCM, and TARGET-LAML, whereas were favorable in KIPAN, KIRC, KIRP, COAD, COADREAD, and READ (Suppl. Fig. S3). In addition, DSS analysis showed that increased *DLAT* was related to poor survival in patients suffering from ACC, BRCA, GBMLGG, LGG, LIHC, PAAD, prostate adenocarcinoma (PRAD), and SKCM, while favorable survival in COAD, COADREAD, and KIRC (Suppl. Fig. S4). Meanwhile, PFI analysis showed that increased *DLAT* was connected with shorter survival in ACC, GBMLGG, LGG, LIHC, and PAAD; while was a protective factor for COAD, COADREAD, and KIRC patients (Suppl. Figs. S5A and S5B). Moreover, DFI analysis showed that PAAD patients had a relatively shorter survival time with high expression levels of *DLAT* (Suppl. Fig. S5C).

**Figure 4:**
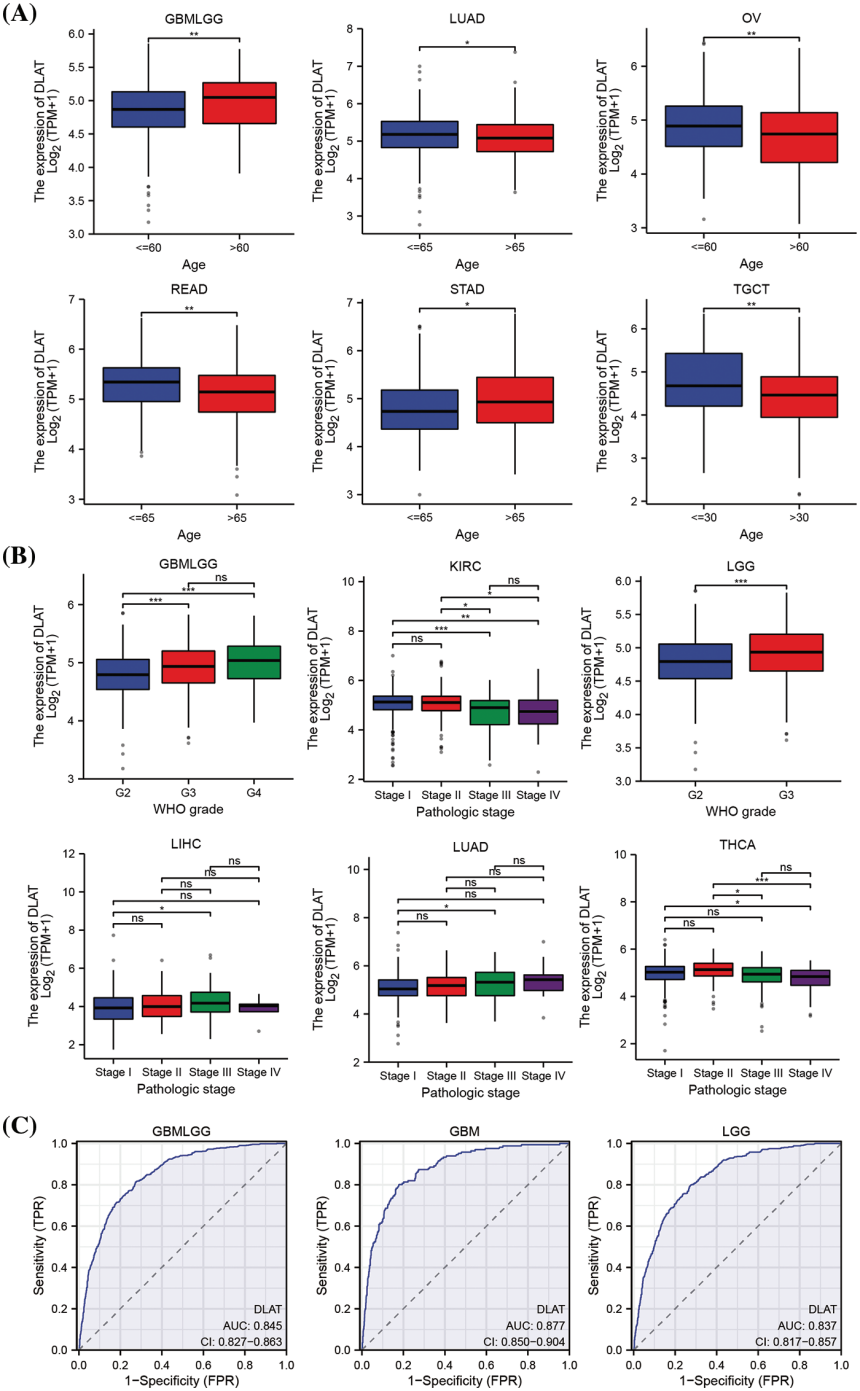
The relationship of *DLAT* to clinical and diagnostic value. (A) Different expressions of *DLAT* in different patients’ age from TCGA. (B) Different expression of *DLAT* on tumor stage from TCGA. (C) ROC curve of *DLAT* expression in the TCGA and GTEx database in GBMLGG, GBM and LGG. ns: not significant; *p < 0.05; **p < 0.01; ***p < 0.001.

We explored the relationship between *DLAT* expression and patient age. We found that older patients had increased *DLAT* expression o in GBMLGG and STAD, while had lower expression in lung adenocarcinoma (LUAD), ovarian serous cystadenocarcinoma (OV), READ, and testicular germ cell tumors (TGCT) than younger patients (Fig. 4A). Moreover, we exhibited that patients who had high DLAT expression had more advanced stages in GBMLGG, LGG, LIHC, and LUAD, while with more favorable stages of KIRC and THCA (Fig. 4B). The diagnostic value of *DLAT* was assessed by ROC curves. The AUC of ROC analysis has relative diagnostic accuracy in GBMLGG (AUC = 0.845), GBM (AUC = 0.877), and LGG (AUC = 0.837) (Fig. 4C). The AUC of ROC analysis had high/relative accuracy (AUC > 0.7) in 18 types of cancers. The detailed results of all cancers are exhibited in Suppl. Table S2. These results suggested that *DLAT* was a good diagnostic factor in most cancers.

**Figure 5:**
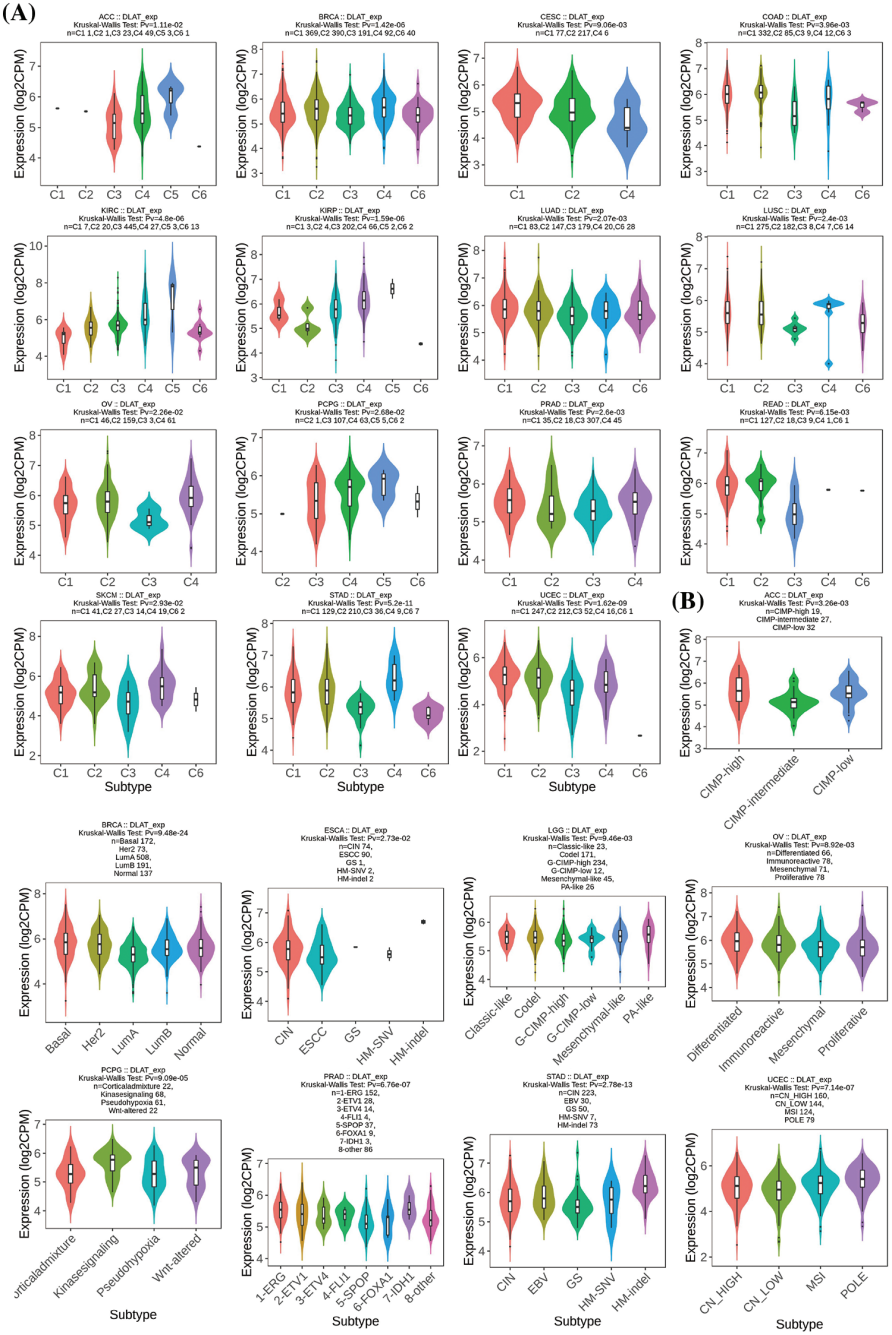
Relationships between *DLAT* expression and (A) immune subtypes and (B) molecular subtypes.

Besides, we demonstrated that *DLAT* was differently expressed in 15 cancer types for immune subtypes, including ACC, BRCA, CESC, COAD, KIRC, KIRP, LUAD, lung squamous cell carcinoma (LUSC), OV, cervical squamous cell carcinoma and endocervical adenocarcinoma (PCPG), PRAD, READ, SKCM, STAD, and UCEC (Fig. 5A), and was differently expressed in 9 cancer types for molecular subtypes, including ACC, BRCA, ESCA, LGG, OV, PCPG, PRAD, STAD, and UCEC (Fig. 5B).

Nowadays, cancer patients often obtain drug resistance, leading to tumor relapse and influencing patients’ prognosis and survival. Therefore, the association between *DLAT* and the drug response of tumor cells was explored to evaluate the therapeutic biomarker value of *DLAT* We exhibited that *DLAT* was positively associated with IC_50_ values of seven compounds, including PI3Kβ inhibitor (AZD6482), PKC inhibitor (midostaurin), HSP90 inhibitor (tanespimycin), and MEK inhibitors (PD0325901, refametinib, trametinib, selumetinib), which suggested that patients with increased DLAT were more resistant to these drugs. However, patients with increased DLAT were more sensitive to 63 compounds, including rTRAIL, HDAC inhibitor (belinostat), and others (Suppl. Table S3). Therefore, the *DLAT* expression may be a biomarker for drug treatment in tumors.

### Tumor microenvironment analysis

**Figure 6:**
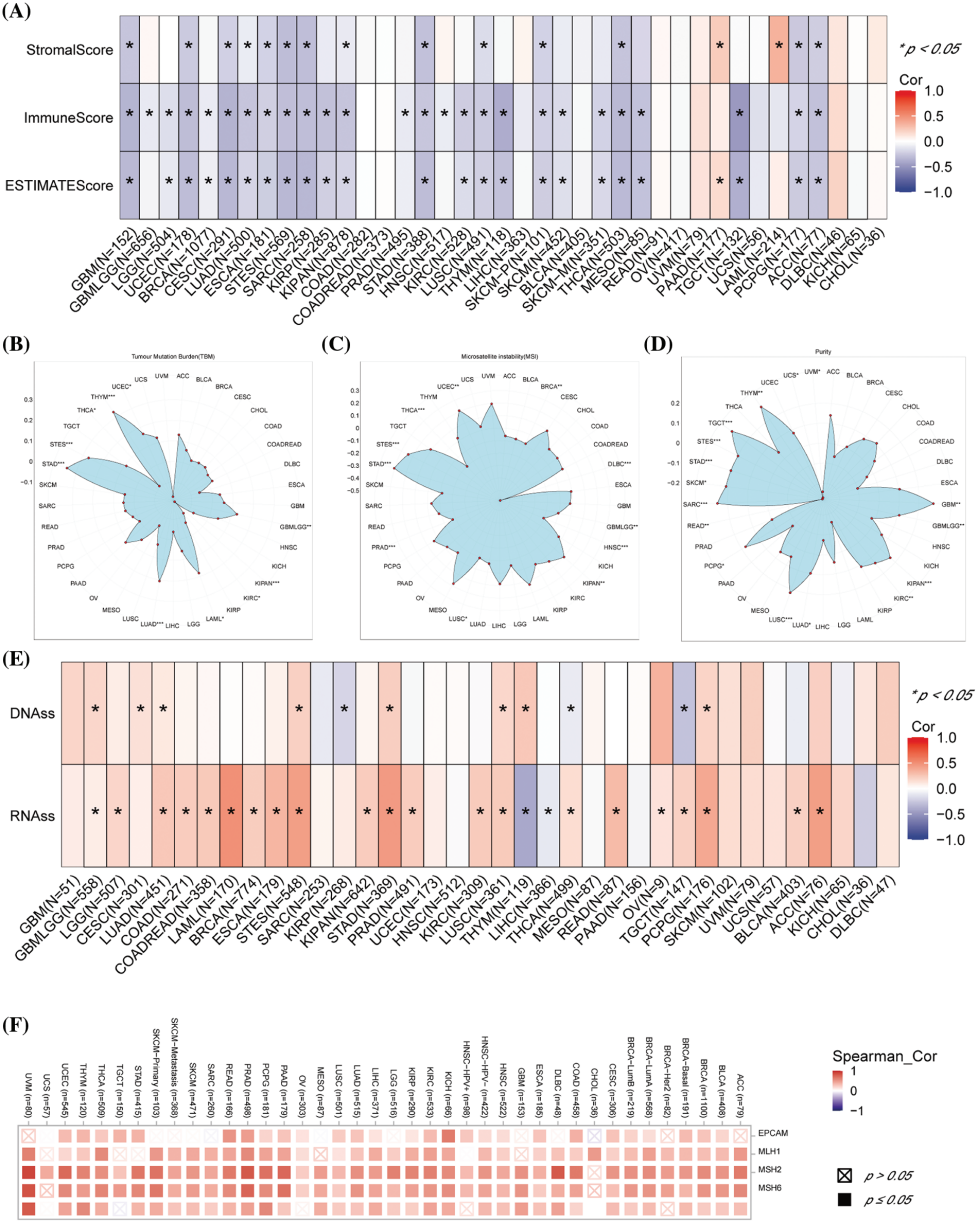
Association between *DLAT* expression and immune infiltration across cancers. (A) Relationship between *DLAT* expression and the StromalScore, ImmuneScore, and ESTIMATEScore. (B) Association of *DLAT* expression with the TBM. (C) Association of *DLAT* expression with MSI. (D) Association of *DLAT* expression with purity. (E) Association of *DLAT* expression with DNAss and RNAss. (F) Association of DLAT expression with MMR. *p < 0.05; **p < 0.01; ***p < 0.001.

The tumor microenvironment (TME) was essential in tumor occurrence and progression. The ESTIMATE algorithm was performed and our results revealed that increased *DLAT* expression had negative scores in GBM, UCEC, CESC, LUAD, ESCA, stomach and esophageal carcinoma (STES), SARC, KIPAN, STAD, LUSC, SKCM-P, THCA, PCPG, and ACC (Fig. 6A).

TMB, MSI, and tumor purity are emerging biomarkers associated with the immunotherapy response. We revealed that *DLAT* expression was positively related to TMB in GBMLGG, LUAD, LAML, STES, STAD, UCES, and THYM (Fig. 6B). In addition, *DLAT* expression was negatively correlated with MSI in GBMLGG, BRCA, PRAD, HNSC, LUSC, THCA, and DLBC (Fig. 6C). Besides, *DLAT* was positively related to purity in GBMLGG, TGCT, THYM, GBM, SARC, LUSC, SKCM, STAD, STES, PCPG, KIPAN, KIRC, and LUAD (Fig. 6D).

RNAss and DNAss can reflect the features of tumor stem cells. The high stemness scores represent the activity of tumor stem cells, are correlated with drug resistance and the continuous proliferation of tumor cells, and are correlated with poorer survival. Additionally, we found *DLAT* was positively correlated with RNAss and DNAss in most cancers, including GBMLGG (Fig. 6E). These results identified *DLAT* as a prognostic factor for patients.

In addition, deficient mismatch repair (MMR) also participated in tumorigenesis and development. We demonstrated that *DLAT* expression was positively correlated with almost all five MMR genes (*MSH2*, *MSH6*, *PMS2*, *MLH1*, and *EPCAM*) in most tumors (Fig. 6F). Therefore, *DLAT* might affect tumor development by regulating the repair of DNA mismatch in cancers.

### Tumor immune microenvironment analysis

**Figure 7:**
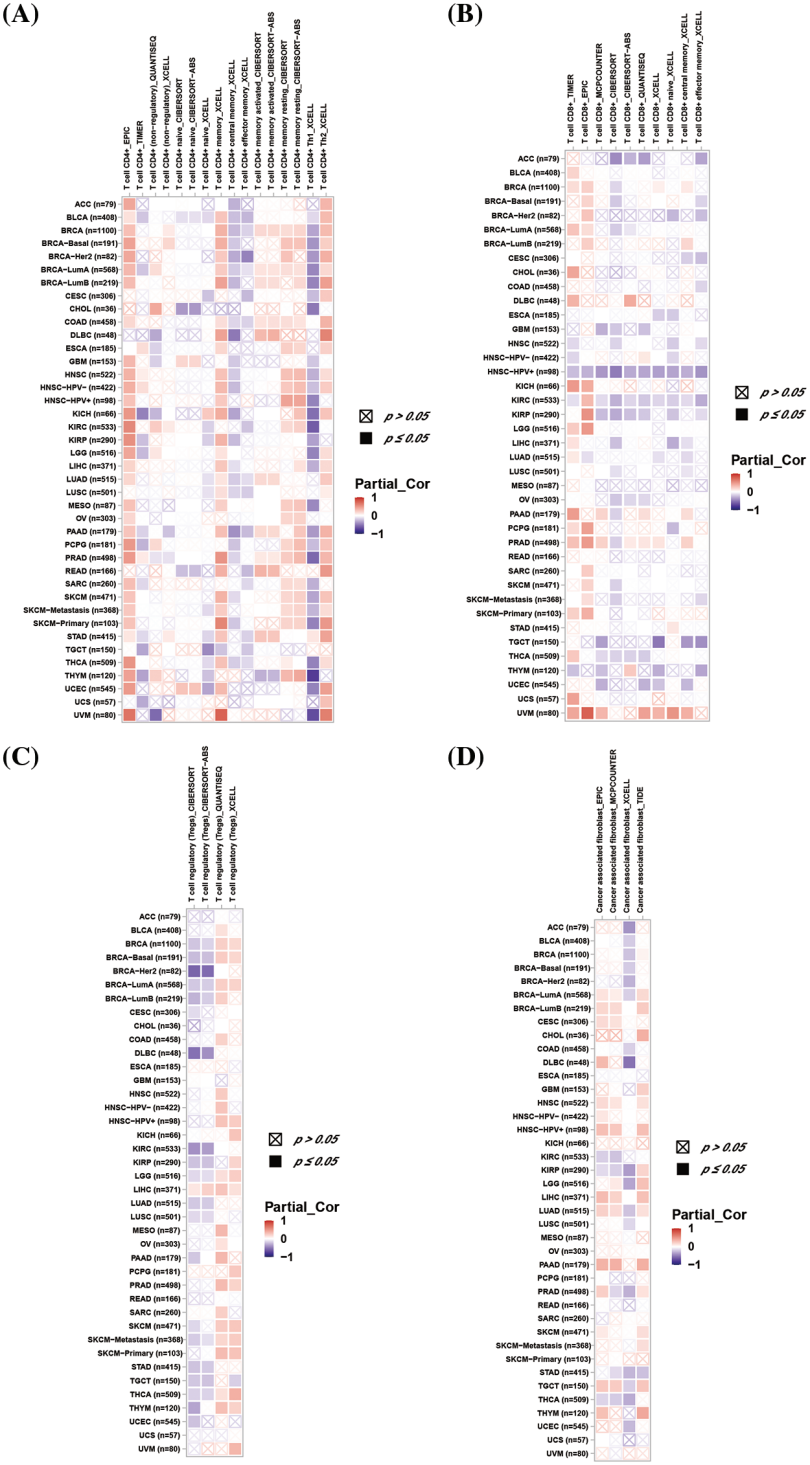
Association between *DLAT* expression and immune cell infiltration across cancers. (A) Relationship between *DLAT* and the immune infiltration of CD4+ T cells. (B) Association between *DLAT* and the immune infiltration of CD8+ T cells. (C) Correlation between *DLAT* and the immune infiltration of Tregs. (D) Relationship between *DLAT* and the immune infiltration of cancer-associated fibroblasts.

We explored the association between *DLAT* expression and immune-related cell infiltration by using different algorithms. *DLAT* expression was statistically negatively associated with CD4+ T cells infiltration in TGCT (Fig. 7A). Moreover, our data demonstrate that *DLAT* expression was statistically negatively related to CD8+ T cells infiltration in ACC, GBM, HNSC, HNSC-HPV+, OV, TGCT, and UCEC (Fig. 7B). In addition, *DLAT* expression was statistically positively correlated in HNSC-HPV+, LIHC, PRAD, and SKCM-P (Fig. 7C). Additionally, *DLAT* expression was positively related in BRCA-lumB, CESC, HNSC, HNSC-HPV+, LIHC, and PAAD (Fig. 7D). Furthermore, we suggested that *DLAT* was significantly negatively associated with the infiltration level of most immune cells across cancers, including CD8+ T cells and plasmacytoid dendritic cells, while positively related to T helper 2 cells and central memory T cells (Suppl. Fig. S6).

**Figure 8:**
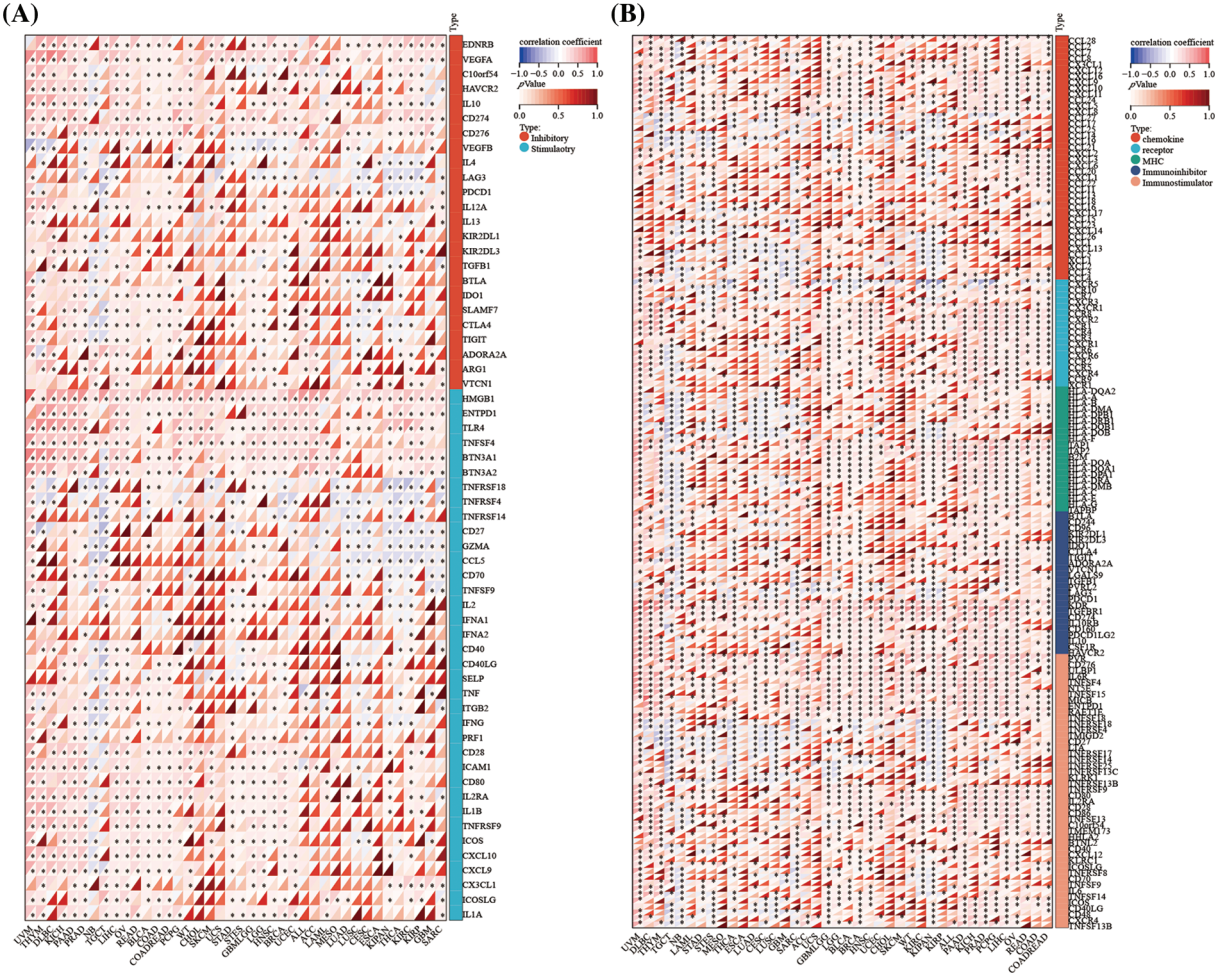
Association between *DLAT* expression and immune-related genes across cancers. (A) Correlation between *DLAT* and immune checkpoint genes. (B) Association between *DLAT* and immune regulatory genes. *p < 0.05.

Tumors can escape immune responses by immune checkpoint proteins and immune regulatory genes could participate in immune response. We exhibited that *DLAT* expression was positively associated with immune checkpoint genes in the majority of tumor types including GBMLGG and LGG (Fig. 8A). Additionally, *DLAT* expression was positively correlated with immune regulatory genes in many tumor types including GBMLGG and LGG (Fig. 8B). In general, these results suggested that *DLAT* might regulate immune cell infiltration and the immune pathways in most tumor types.

### Enrichment analysis

**Figure 9:**
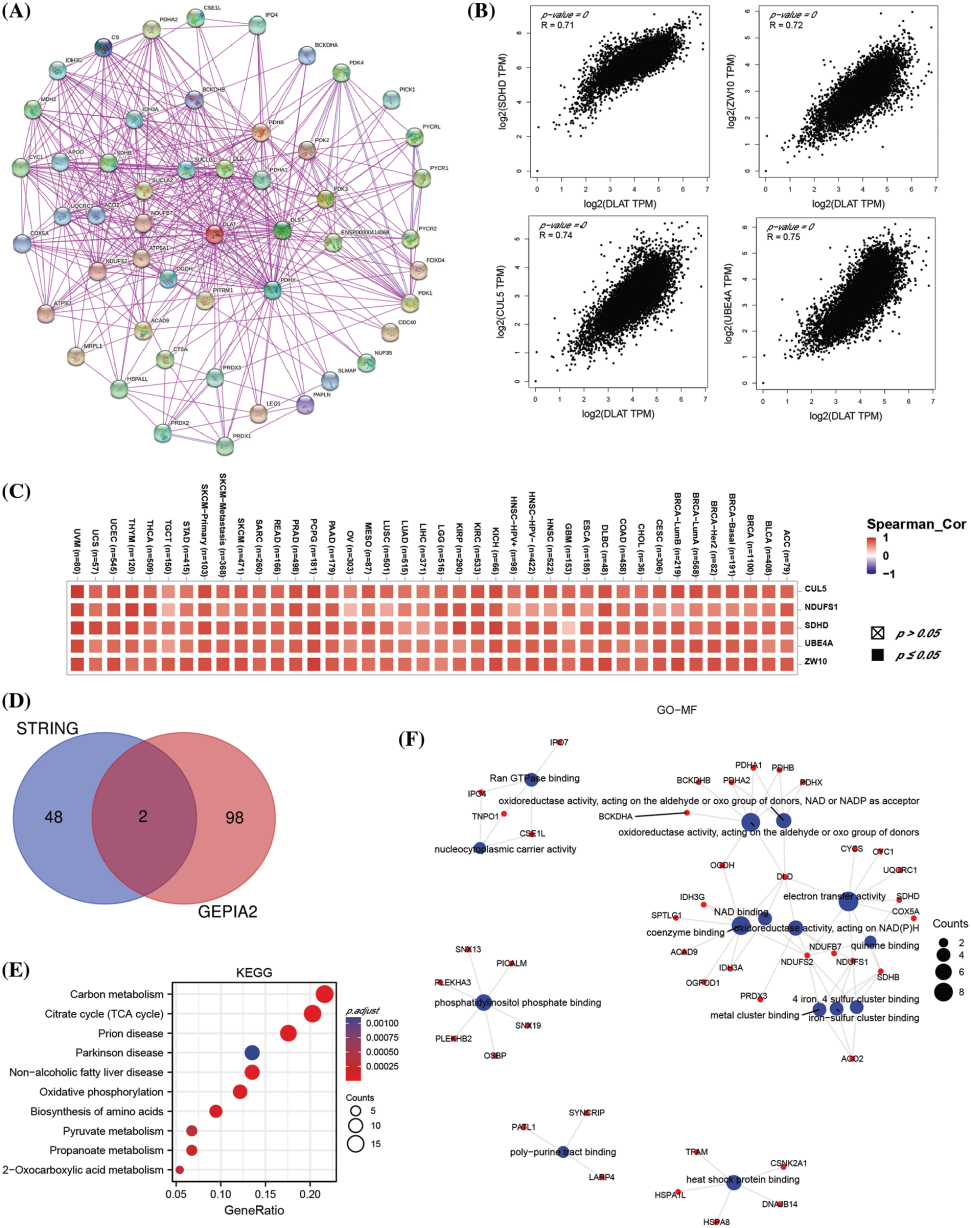
The enrichment analysis of *DLAT*-related genes across cancers. (A) The *DLAT*-binding proteins from the STRING. (B) The top 4 *DLAT*-related genes by GEPIA2. (C) Heatmap for the five *DLAT*-related genes across cancers. (D) Venn plot for the intersection of the *DLAT*-binding and related genes. (E) KEGG pathway analysis from the two datasets. (F) The circle map for the molecular function in GO analysis.

A total of 50 *DLAT*-binding proteins were acquired by using the STRING tool (Fig. 9A). The top 100 *DLAT*-related genes were obtained from the GEPIA2 tool. We identified that the *DLAT* was positively correlated with Succinate Dehydrogenase Complex Subunit D (*SDHD*), Zw10 Kinetochore Protein (*ZW10*), Cullin 5 (*CUL5*), Ubiquitination Factor E4A (*UBE4A*) and NADH: Ubiquinone Oxidoreductase Core Subunit S1 (*NDUFS1*) (Fig. 9B). The heatmap data also exhibited a positive relationship between *DLAT* and the above five genes across cancer (Fig. 9C). Dihydrolipoamide dehydrogenase (DLD) and citrate synthase (CS) were two common genes in these two groups (Fig. 9D). KEGG pathway enrichment analysis of these two datasets revealed that these genes were mainly related to carbon metabolism, the citrate cycle (TCA cycle), and others (Fig. 9E). GO enrichment analysis identified that these genes mainly played molecular functions in oxidoreductase activity, electron transfer activity, and others (Fig. 9F). Moreover, we performed GSEA analysis and found that DLAT related genes were mainly enriched in pathways like cell cycle in BLCA and GBMLGG (Suppl. Fig. S7).

### DLAT acts as a biomarker for glioma cancer

**Figure 10:**
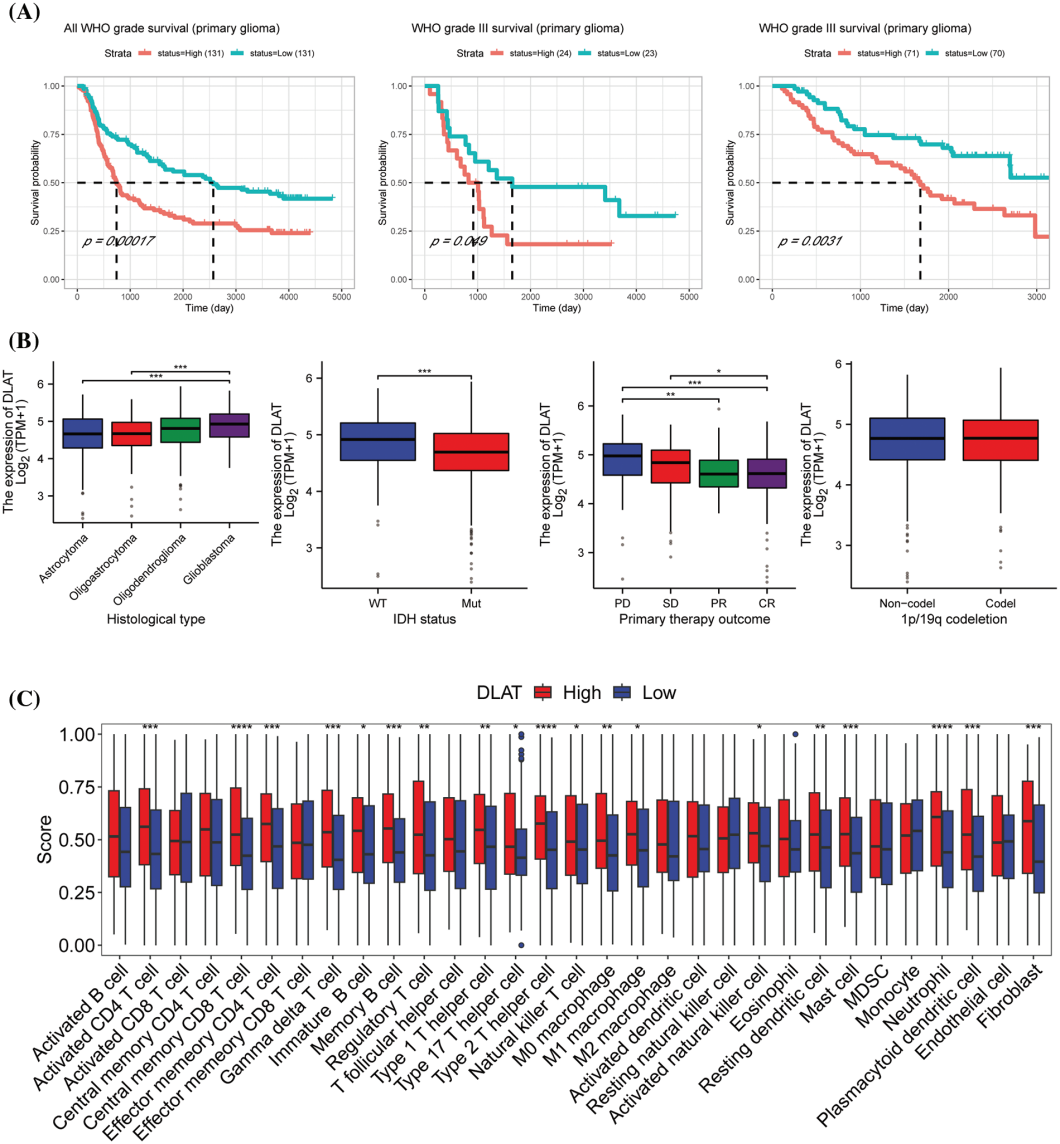
The clinical value and the potent biological functions of DLAT in glioma. (A) The survival analysis of DLAT in glioma patients. (B) Relationship between DLAT expression and clinical characteristics in GBMLGG, including histological type, IDH status, primary therapy outcome, and p/19q codeletion. (C) Difference of the infiltration of immune cells between high and low DLAT expression groups in CGGA database. *p < 0.05; **p < 0.01; ***p < 0.001; ****p < 0.0001.

The above studies demonstrated that *DLAT* was significantly highly expressed in GBM and LGG, and it was strongly associated with OS, DSS, and PFI in GBMLGG patients. Besides *DLAT* expression was also significantly associated with tumor microenvironment. Therefore, we next explored the clinical value and the potent biological functions of *DLAT* in glioma patients. We identified that the protein levels of *DLAT* were increased in glioma tissue than in normal tissue, especially in high-grade glioma patients (Suppl. Fig. S8). We assessed the prognostic value of *DLAT* in CGGA clinical samples, and we showed that increased *DLAT* expression was related to poor survival in primary glioma patients, and in grade III patients in different datasets (Fig. 10A). Furthermore, we found that higher *DLAT* levels were correlated with glioblastoma, IDH status (wild type), and primary therapy outcome (PD) (Fig. 10B). While *DLAT* expression was not correlated with 1p/19q codeletion status (Fig. 10B).

Then we performed GSEA to explore the functions of *DLAT* in GBMLGG. The top 10 GSEA terms in the indicated tumor types are shown in Fig. 10C. We demonstrated that *DLAT* had a strong association with sister chromatid segregation, and ATP-dependent activity acting on DNA, mainly located in the synaptic membrane. The enrichment HALLMARK pathways showed that *DLAT* might play a role in the G2/M checkpoint, E2F targets, Uv response, epithelial-mesenchymal transition (EMT), and mitotic spindles (Suppl. Fig. S9). These suggested *DLAT* may participate in the cell cycle to affect the occurrence and progression of tumors. Furthermore, we performed an experimental study to verify the function of *DLAT* in glioma cells.

Furthermore, we performed ssGSEA analysis to evaluate immune cell infiltrtions in glioma. We discovered that DLAT high expression group was immune-active and stroma-rich subtype, which had high infiltrating levels of M0 and M1 macrophages, fibroblast, actived CD4 T cell, central memory CD8 T cell, effector memory CD4 Tcell, gamma delta T cell, immature B cell, memory B cell, regulatory T cell, type 1, 17, and 2 helper T cells, actived natural killer cell, mast cell, matural killer T cell, neutrophil, and plasmacytoid dendritic cell (Fig. 10C and Suppl. Fig. S10). While low DLAT expression group was immune-desert subtype, was characterized by low infiltration of most immune and stromal cells.

**Figure 11:**
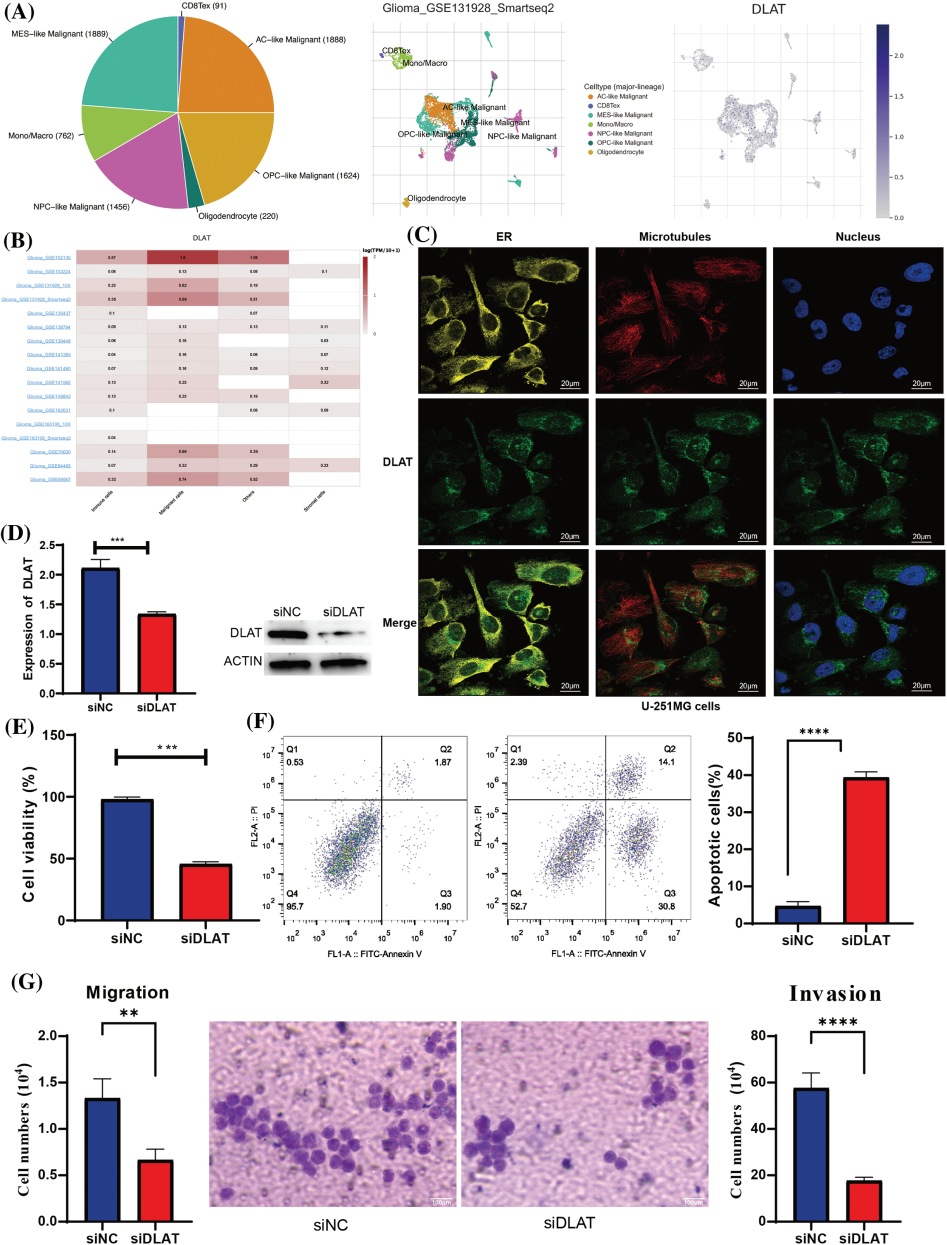
Validation of the expression of the *DLAT*. (A) The expression profiles of *DLAT* in the single cells from glioma tissues. (B) *DLAT* RNA-seq analysis of the Genotype-Tissue Expression (GTEx) and TCGA sample set. (C) The qRT-PCR and WB detected the efficacy of silencing *DLAT* in A172 cells. (D) The silencing of *DLAT* inhibited the cell viability of A172 cells. (E) The silencing of *DLAT* promoted the apoptosis of the A172 cells. (F, G) Migration and invasion ratio of the GBM cell line by Transwell membranes (5-mm pore size). Independent experiments were performed 3 times. n = 3 per group. **p < 0.01; ***p < 0.001; ****p < 0.0001.

*DLAT* was mainly expressed in malignant cells at single cells in glioma tissues by TISCH (Figs. 11A and 11B). Besides, we found that *DLAT* was mainly located in the endoplasmic reticulum (ER) (Fig. 11C). To validate the function of *DLAT* in glioma, we developed silenced *DLAT* A172 glioma cell lines (Fig. 11D). Furthermore, *DLAT* silencing inhibited cell proliferation (Fig. 11E), promoted cell apoptosis (Fig. 11F), and inhibited the migration and invasion (Fig. 11G). These results demonstrated that *DLAT* was essential in the occurrence and development of glioma.

## Discussion

*DLAT* is subunit E2 of the pyruvate dehydrogenase complex (PDC) [4], and was essential for malignancies by regulating cuproptosis [24]. *DLAT* was reported to be associated with some cancers and participated in the occurrence and prognosis of tumors. However, the detailed role of *DLAT* across cancers and the potential mechanism for tumorigenesis are still unclear. Therefore, we performed a comprehensive analysis of *DLAT* across cancers and validated its function in glioma.

We demonstrated that the *DLAT* gene was increased in most cancers, and was a risk prognostic factor. In addition, *DLAT* expression was associated with age, tumor stage, and diagnostic value. These results were consistent with previous studies [6,10,12–14]. *DLAT* could also affect the efficacy of many compounds. A previous study showed that alternate killed prostate cancer by targeting the *DLAT* protein [25]. This indicated that *DLAT* acted as a biomarker for the prognosis, diagnosis, and therapy of cancer patients.

Genetic and epigenetic alterations have been closely associated with tumorigenesis. RNA methylation modifications play a role in many processes, like cell differentiation and development [16,26–28]. DNA methylation plays an important role in the occurrence and progression of cancers [29,30]. Our results identified that *DLAT* was positively related to RNA methylation modification genes and DNA methylation. These revealed that genetic and epigenetic alterations could affect *DLAT* expression and participate in the development of tumors.

TMB, MSI, and tumor purity are emerging biomarkers associated with the immunotherapy response [18,31,32]. Moreover, previous studies also clarified that high somatic TMB was associated with favorable survival prognosis in cancer patients with immunotherapy [33,34]. Additionally, patients with gastroesophageal cancer and colorectal cancer with high-frequency MSI were related to favorable efficacy and good survival after immunotherapy [34,35]. RNAss and DNAss can reflect the features of tumor stem cells [20]. The high stemness scores represent the activity of tumor stem cells, and is associated with drug resistance and the continuous proliferation of tumor cells, and are correlated with poorer survival [21]. MMR helps cells to maintain genomic stability. MMR deficiency is associated with the therapeutic efficacy of immunotherapy [36]. We showed that *DLAT* expression was significantly associated with TMB, MSI, purity, stemness scores, and MMR in multiple cancer types, thereby affecting the efficacy of immunotherapy. Therefore, *DLAT* could be a possible immunotherapeutic target for cancers.

Currently, the tumor immune microenvironment (TIME), comprising various infiltrating immune and stromal cells influences malignancies, including the proliferation and invasion of tumors [37,38], and affects treatment response and clinical outcomes [39]. The ESTIMATE algorithm is a prognostic factor in cancers [40]. The high ESTIMATE score is associated with a low purity, advanced cancer stage, and poor prognosis [40,41]. The present research displayed notable negative correlations between *DLAT* expression and all three scores, for example in GBM, which may explain, in some ways, the essential role of *DLAT* in GBM, as mentioned above. The tumor stroma contains immune infiltration cells, which critically take part in tumor occurrence and progression [38,42]. Our study exhibited that *DLAT* expression was associated with CD4+ T cells, CD8+ T cells, Tregs, and cancer-associated fibroblasts in many cancers. For example, CD8+ T cells were negatively correlated with GBM. Furthermore, *DLAT* expression was positively related to different immune-related genes and immune infiltrating cells in cancers such as GBM, UVM, and DLBC. Therefore, we inferred that *DLAT* might form positive feedback with certain immune checkpoint genes, inhibiting the function of cytotoxic immune cells, enabling tumor cells to escape immune surveillance, and enhancing their malignancy.

Single-cell analysis displayed that *DLAT* might participate in cancers by regulating DNA repair and stemness. KEGG and GO enrichment analyses suggested that *DLAT* was related to the TCA cycle, and others. GSEA suggested that *DLAT* participated in the processes of the cell cycle, transcription factors, inflammatory response, and so on. In conclusion, these results identified that *DLAT* played an oncogenic role across cancers.

In addition, we analyzed that *DLAT* was highly expressed in glioma patients, and acted as a risk prognostic factor. Besides, the high DLAT expression had immune-active and stroma-rich subtypes, which had both tumor-suppressing and tumor-promoting immune cells and stromal cells, might benefit from immunotherapy [22]. This result was consistent with the result of relationship between DLAT and MSI status. However, these results only reflected the relationship but not the causation, more experiments are needed. *DLAT* silencing experiment demonstrated that *DLAT* was essential in the occurrence and development of glioma.

The above comprehensive analysis identified that *DLAT* might be a potential prognostic and immune infiltration marker and therapeutic target for tumors, while further experiments are needed to validate its correlation with immune infiltration, and verify the relationship with the current immunotherapy. Undeniably, our study had several limitations. First, part of our study was based on public databases, and more clinical studies were needed to understand the potential mechanism further to validate the relationships. Second, more experimental studies are needed to further understand the potential mechanism.

## Conclusion

In summary, our study systematically performed a multi-omics combined analysis of *DLAT* across cancers, and we demonstrated the abnormal expression profiles of *DLAT* and its relationship with clinical, prognosis, epigenetic alterations, and immune response. Additionally, we also analyzed the potential function and mechanism of *DLAT* in a variety of human cancers, and validated its oncogenic role in glioma cells.

## Supplementary Materials

Figure S1 **DLAT gene expression in pan-cancer**. **(A)** DLAT expression in normal samples from GTEx database. **(B)** DLAT gene expression in cancer cell lines from CCLE database. **(C)** DLAT gene expression in cancers from TCGA database.

Figure S2 **The genetic alteration analysis of DLAT in pan-cancer**. **(A)** The alteration frequency with different types of mutations. **(B)** Mutation sites. **(C)** The 3D structure of DLAT.

Figure S3 **Kaplan‒Meier curves showing the relationships of DLAT expression with OS**. **(A)** DLAT was a risk prognostic factor in ACC, BRCA, GBMLGG, LGG, LIHC, PAAD, SKCM and TARGET-LAML. **(B)** DLAT was a favorable prognostic factor in KIPAN, KIRC, KIRP, COAD, COADREAD and READ.

Figure S4 **Kaplan‒Meier curves showing the relationships of DLAT expression with DSS**. **(A)** DLAT was a risk prognostic factor in ACC, BRCA, GBMLGG, LGG, LIHC, PAAD, PRAD and SKCM. **(B)** DLAT was a favorable prognostic factor in COAD, COADREAD and KIRC.

Figure S5 **Kaplan‒Meier curves showing the relationships of DLAT expression with PFI (A, B) and DFI **(C)****. **(A)** For PFI analysis, DLAT was a risk prognostic factor in ACC, GBMLGG, LGG, LIHC and PAAD. **(B)** For PFI analysis, DLAT was a favorable prognostic factor in COAD, COADREAD and KIRC. **(C)** For DFI analysis, DLAT was a risk prognostic factor in PAAD.

Figure S6 Correlation of DLAT with the level of 28 type immune infiltrating cells. *p<0.05

Figure S7 The HALLMARK enrichment analysis of DLAT related genes in BLCA and GBMLGG by GSEA.

Figure S8 The immunohistochemical staining of DLAT protein in glioma patients’ tissue and normal tissue.

Figure S9 GO term and HALLMARK pathway analysis in GBMLGG.

Figure S10 Heatmap illustrating the infiltration of 31 TME cells in CGGA glioma samples.

## Data Availability

The datasets presented in this study can be found in online repositories.

## Supplementary Materials

The supplementary material is available online at https://doi.org/10.32604/or.2024.048138.

## References

[1] Singh, A. K., McGuirk, J. P. (2020). CAR T cells: Continuation in a revolution of immunotherapy. The Lancet Oncology*,* 21 *(*3*),* e168–e178. 10.1016/s1470-2045(19)30823-x; 32135120

[2] He, X., Xu, C. (2020). Immune checkpoint signaling and cancer immunotherapy. Cell Research*,* 30 *(*8*),* 660–669. 10.1038/s41422-020-0343-4; 32467592 PMC7395714

[3] Ribas, A., Wolchok, J. D. (2018). Cancer immunotherapy using checkpoint blockade. Science*,* 359 *(*6382*),* 1350–1355. 10.1126/science.aar4060; 29567705 PMC7391259

[4] Stacpoole, P. W. (2017). Therapeutic targeting of the pyruvate dehydrogenase complex/pyruvate dehydrogenase kinase (PDC/PDK) axis in cancer. Journal of the National Cancer Institute*,* 109 *(*11*)*. 10.1093/jnci/djx071; 29059435

[5] Houten, S. M., Wanders, R. J. A. (2010). A general introduction to the biochemistry of mitochondrial fatty acid β-oxidation. Journal of Inherited Metabolic Disease*,* 33 *(*5*),* 469–477. 10.1007/s10545-010-9061-2; 20195903 PMC2950079

[6] Goh, W. Q., Ow, G. S., Kuznetsov, V. A., Chong, S., Lim, Y. P. (2015). DLAT subunit of the pyruvate dehydrogenase complex is upregulated in gastric cancer-implications in cancer therapy. American Journal of Translational Research*,* 7 *(*6*),* 1140–1151; 26279757 PMC4532746

[7] Hanahan, D., Weinberg, R. A. (2011). Hallmarks of cancer: The next generation. Cell*,* 144 *(*5*),* 646–674. 10.1016/j.cell.2011.02.013; 21376230

[8] Pavlova, N. N., Thompson, C. B. (2016). The emerging hallmarks of cancer metabolism. Cell Metabolism*,* 23 *(*1*),* 27–47. 10.1016/j.cmet.2015.12.006; 26771115 PMC4715268

[9] Ward, P. S., Thompson, C. B. (2012). Metabolic reprogramming: A cancer hallmark even Warburg did not anticipate. Cancer Cell*,* 21 *(*3*),* 297–308. 10.1016/j.ccr.2012.02.014; 22439925 PMC3311998

[10] Chen, Q., Wang, Y., Yang, L., Sun, L., Wen, Y. et al. (2022). PM2.5 promotes NSCLC carcinogenesis through translationally and transcriptionally activating DLAT-mediated glycolysis reprograming. Journal of Experimental & Clinical Cancer Research*,* 41 *(*1*),* 229. 10.1186/s13046-022-02437-8; 35869499 PMC9308224

[11] Yang, M., Zheng, H., Xu, K., Yuan, Q., Aihaiti, Y. et al. (2022). A novel signature to guide osteosarcoma prognosis and immune microenvironment: Cuproptosis-related lncRNA. Frontiers in immunology*,* 13*,* 919231. 10.3389/fimmu.2022.919231; 35967366 PMC9373797

[12] Bian, Z., Fan, R., Xie, L. (2022). A novel cuproptosis-related prognostic gene signature and validation of differential expression in clear cell renal cell carcinoma. Genes*,* 13 *(*5*),* 851. 10.3390/genes13050851; 35627236 PMC9141858

[13] Li, L., Li, L., Sun, Q. (2022). High expression of cuproptosis-related SLC31A1 gene in relation to unfavorable outcome and deregulated immune cell infiltration in breast cancer: An analysis based on public databases. BMC Bioinformatics*,* 23 *(*1*),* 350. 10.1186/s12859-022-04894-6; 35996075 PMC9394027

[14] Chen, S., Cao, G., Wu, W., Lu, Y., He, X. et al. (2020). Mining novel cell glycolysis related gene markers that can predict the survival of colon adenocarcinoma patients. Bioscience Reports*,* 40 *(*8*)*. 10.1042/bsr20201427; 32744303 PMC7426632

[15] Liu, J., Lichtenberg, T., Hoadley, K. A., Poisson, L. M., Lazar, A. J. et al. (2018). An integrated TCGA pan-cancer clinical data resource to drive high-quality survival outcome analytics. Cell*,* 173 *(*2*),* 400–416.E11. 10.1016/j.cell.2018.02.052; 29625055 PMC6066282

[16] Zhao, B. S., Roundtree, I. A., He, C. (2017). Post-transcriptional gene regulation by mRNA modifications. Nature Reviews Molecular Cell Biology*,* 18 *(*1*),* 31–42. 10.1038/nrm.2016.132; 27808276 PMC5167638

[17] Li, R., Han, D., Shi, J., Han, Y., Tan, P. et al. (2020). Choosing tumor mutational burden wisely for immunotherapy: A hard road to explore. Biochimica et Biophysica Acta Reviews on Cancer*,* 1874 *(*2*),* 188420. 10.1016/j.bbcan.2020.188420; 32828886

[18] Choucair, K., Morand, S., Stanbery, L., Edelman, G., Dworkin, L. et al. (2020). TMB: A promising immune-response biomarker, and potential spearhead in advancing targeted therapy trials. Cancer Gene Therapy*,* 27 *(*12*),* 841–853. 10.1038/s41417-020-0174-y; 32341410

[19] Bonneville, R., Krook, M. A., Kautto, E. A., Miya, J., Wing, M. R. et al. (2017). Landscape of microsatellite instability across 39 cancer types. JCO Precision Oncology*,* 1*,* 1–15. 10.1200/po.17.00073; 29850653 PMC5972025

[20] Thorsson, V., Gibbs, D. L., Brown, S. D., Wolf, D., Bortone, D. S. et al. (2018). The immune landscape of cancer. Immunity*,* 48 *(*4*),* 812–830.E14. 10.1016/j.immuni.2018.03.023; 29628290 PMC5982584

[21] Malta, T. M., Sokolov, A., Gentles, A. J., Burzykowski, T., Poisson, L. et al. (2018). Machine learning identifies stemness features associated with oncogenic dedifferentiation. Cell*,* 173 *(*2*),* 338–354.E15. 10.1016/j.cell.2018.03.034; 29625051 PMC5902191

[22] Mao, Y., Xu, Y., Chang, J., Chang, W., Lv, Y. et al. (2022). The immune phenotypes and different immune escape mechanisms in colorectal cancer. Frontiers in Immunology*,* 13*,* 968089. 10.3389/fimmu.2022.968089; 36032084 PMC9399611

[23] Yuan, H., Yan, M., Zhang, G., Liu, W., Deng, C. et al. (2019). CancerSEA: A cancer single-cell state atlas. Nucleic Acids Research*,* 47 *(*D1*),* D900–D908. 10.1093/nar/gky939; 30329142 PMC6324047

[24] Tsvetkov, P., Coy, S., Petrova, B., Dreishpoon, M., Verma, A. et al. (2022). Copper induces cell death by targeting lipoylated TCA cycle proteins. Science*,* 375 *(*6586*),* 1254–1261. 10.1126/science.abf0529; 35298263 PMC9273333

[25] Li, C., He, C., Xu, Y., Xu, H., Tang, Y. et al. (2019). Alternol eliminates excessive ATP production by disturbing Krebs cycle in prostate cancer. The Prostate*,* 79 *(*6*),* 628–639. 10.1002/pros.23767; 30663084 PMC6644699

[26] Fernandez Rodriguez, G., Cesaro, B., Fatica, A. (2022). Multiple roles of m6A RNA modification in translational regulation in cancer. International Journal of Molecular Sciences*,* 23 *(*16*),* 8971. 10.3390/ijms23168971; 36012237 PMC9408962

[27] Qu, X., Zhang, Y., Sang, X., Ren, D., Zhao, H. et al. (2022). Methyladenosine modification in RNAs: From regulatory roles to therapeutic implications in cancer. Cancers*,* 14 *(*13*),* 3195. 10.3390/cancers14133195; 35804965 PMC9264946

[28] Li, M., Tao, Z., Zhao, Y., Li, L., Zheng, J. et al. (2022). 5-methylcytosine RNA methyltransferases and their potential roles in cancer. Journal of Translational Medicine*,* 20 *(*1*),* 214. 10.1186/s12967-022-03427-2; 35562754 PMC9102922

[29] Klutstein, M., Nejman, D., Greenfield, R., Cedar, H. (2016). DNA methylation in cancer and aging. Cancer Research*,* 76 *(*12*),* 3446–3450. 10.1158/0008-5472.can-15-3278; 27256564

[30] Jones, P. A., Baylin, S. B. (2007). The epigenomics of cancer. Cell*,* 128 *(*4*),* 683–692. 10.1016/j.cell.2007.01.029; 17320506 PMC3894624

[31] Fumet, J. D., Truntzer, C., Yarchoan, M., Ghiringhelli, F. (2020). Tumour mutational burden as a biomarker for immunotherapy: Current data and emerging concepts. European Journal of Cancer*,* 131*,* 40–50. 10.1016/j.ejca.2020.02.038; 32278982 PMC9473693

[32] Dudley, J. C., Lin, M. T., Le, D. T., Eshleman, J. R. (2016). Microsatellite instability as a biomarker for PD-1 blockade. Clinical Cancer Research*,* 22 *(*4*),* 813–820. 10.1158/1078-0432.ccr-15-1678; 26880610

[33] Liu, L., Bai, X., Wang, J., Tang, X. R., Wu, D. H. et al. (2019). Combination of TMB and CNA stratifies prognostic and predictive responses to immunotherapy across metastatic cancer. Clinical Cancer Research*,* 25 *(*24*),* 7413–7423. 10.1158/1078-0432.ccr-19-0558; 31515453

[34] Samstein, R. M., Lee, C. H., Shoushtari, A. N., Hellmann, M. D., Shen, R. et al. (2019). Tumor mutational load predicts survival after immunotherapy across multiple cancer types. Nature Genetics*,* 51 *(*2*),* 202–206. 10.1038/s41588-018-0312-8; 30643254 PMC6365097

[35] van Velzen, M. J. M., Derks, S., van Grieken, N. C. T., Haj Mohammad, N., van Laarhoven, H. W. M. (2020). MSI as a predictive factor for treatment outcome of gastroesophageal adenocarcinoma. Cancer Treatment Reviews*,* 86*,* 102024. 10.1016/j.ctrv.2020.102024; 32388292

[36] He, Y., Zhang, L., Zhou, R., Wang, Y., Chen, H. (2022). The role of DNA mismatch repair in immunotherapy of human cancer. International Journal of Biological Sciences*,* 18 *(*7*),* 2821–2832. 10.7150/ijbs.71714; 35541922 PMC9066103

[37] Binnewies, M., Roberts, E. W., Kersten, K., Chan, V., Fearon, D. F. et al. (2018). Understanding the tumor immune microenvironment (TIME) for effective therapy. Nature Medicine*,* 24 *(*5*),* 541–550. 10.1038/s41591-018-0014-x; 29686425 PMC5998822

[38] Hinshaw, D. C., Shevde, L. A. (2019). The tumor microenvironment innately modulates cancer progression. Cancer Research*,* 79 *(*18*),* 4557–4566. 10.1158/0008-5472.can-18-3962; 31350295 PMC6744958

[39] Wu, T., Dai, Y. (2017). Tumor microenvironment and therapeutic response. Cancer Letters*,* 387*,* 61–68. 10.1016/j.canlet.2016.01.043; 26845449

[40] Yoshihara, K., Shahmoradgoli, M., Martínez, E., Vegesna, R., Kim, H. et al. (2013). Inferring tumour purity and stromal and immune cell admixture from expression data. Nature Communications*,* 4*,* 2612. 10.1038/ncomms3612; 24113773 PMC3826632

[41] Aran, D., Sirota, M., Butte, A. J. (2015). Systematic pan-cancer analysis of tumour purity. Nature Communications*,* 6*,* 8971. 10.1038/ncomms9971; 26634437 PMC4671203

[42] Lei, X., Lei, Y., Li, J. K., Du, W. X., Li, R. G. et al. (2020). Immune cells within the tumor microenvironment: Biological functions and roles in cancer immunotherapy. Cancer Letters*,* 470*,* 126–133. 10.1016/j.canlet.2019.11.009; 31730903

