# Supplementary figures and images for: A comprehensive and systematic analysis of Dihydrolipoamide S-acetyltransferase (DLAT) as a novel prognostic biomarker in pan-cancer and glioma
Source: Oncol Res. 2024 Nov 13;32(12):1903–19. doi: 10.32604/or.2024.048138 (PMC11576973; doi:10.32604/or.2024.048138)

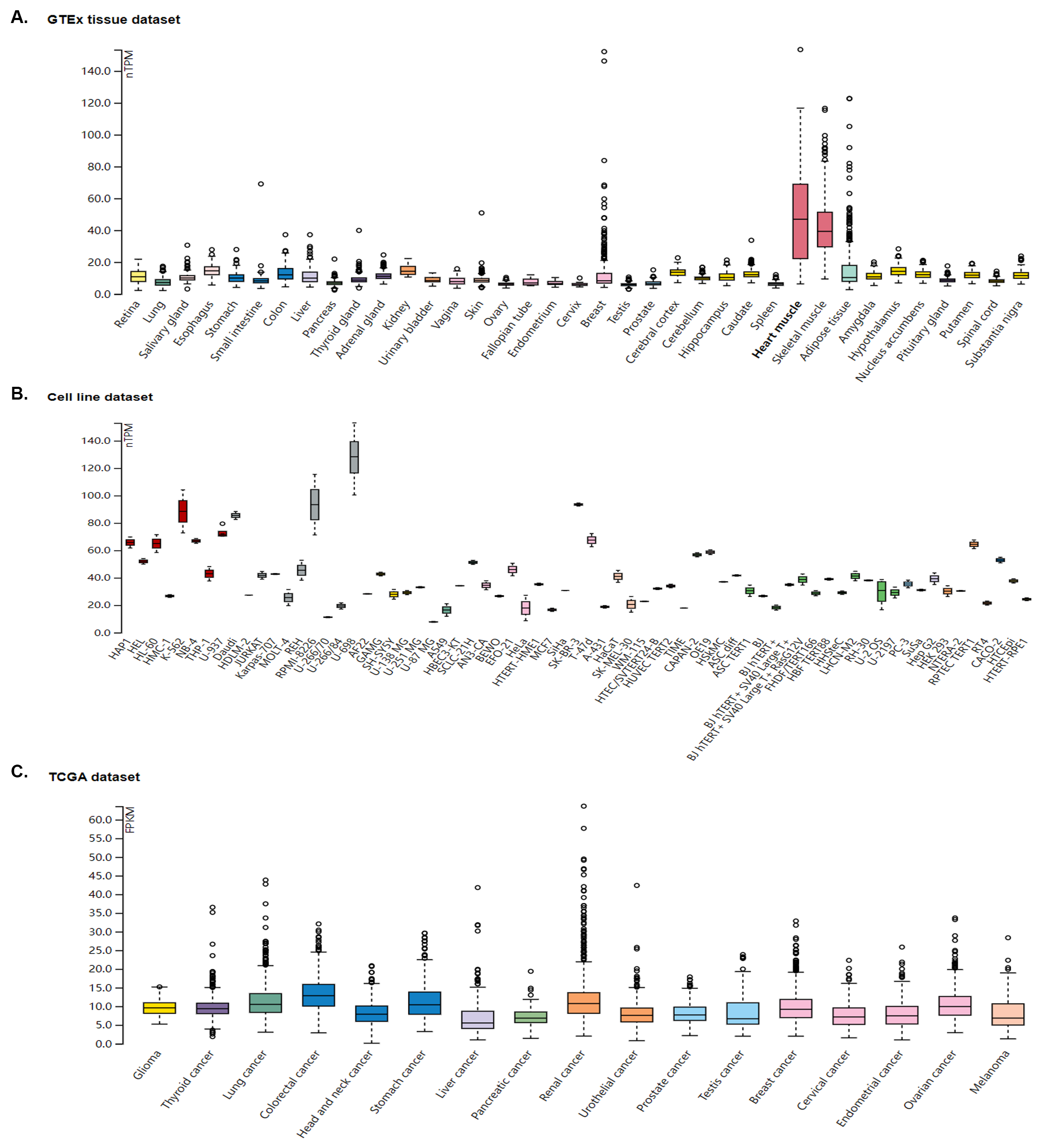

Supplement: Figure S1 [file OncolRes-32-48138-s001.tif]

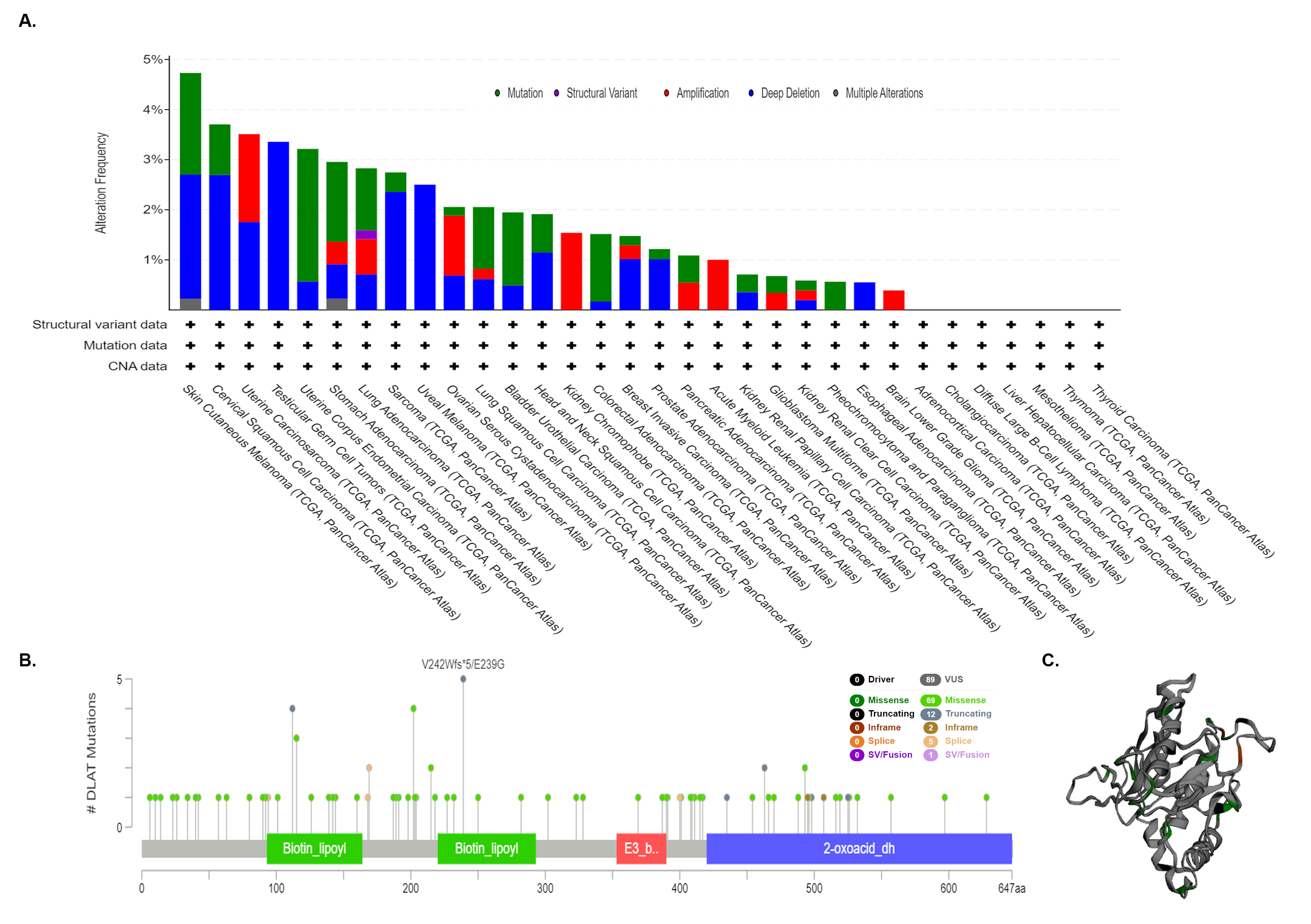

Supplement: Figure S2 [file OncolRes-32-48138-s002.tif]

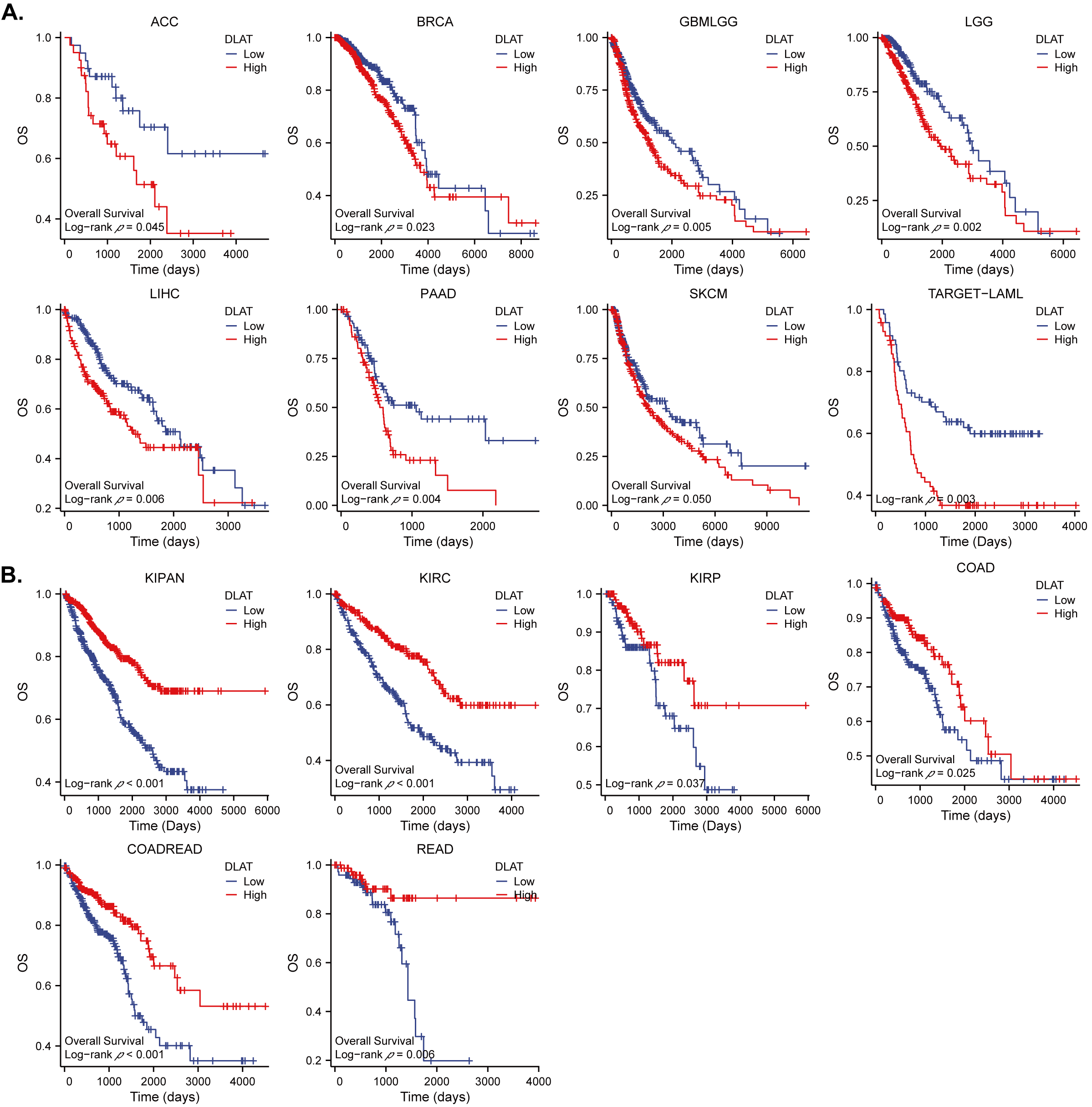

Supplement: Figure S3 [file OncolRes-32-48138-s003.tif]

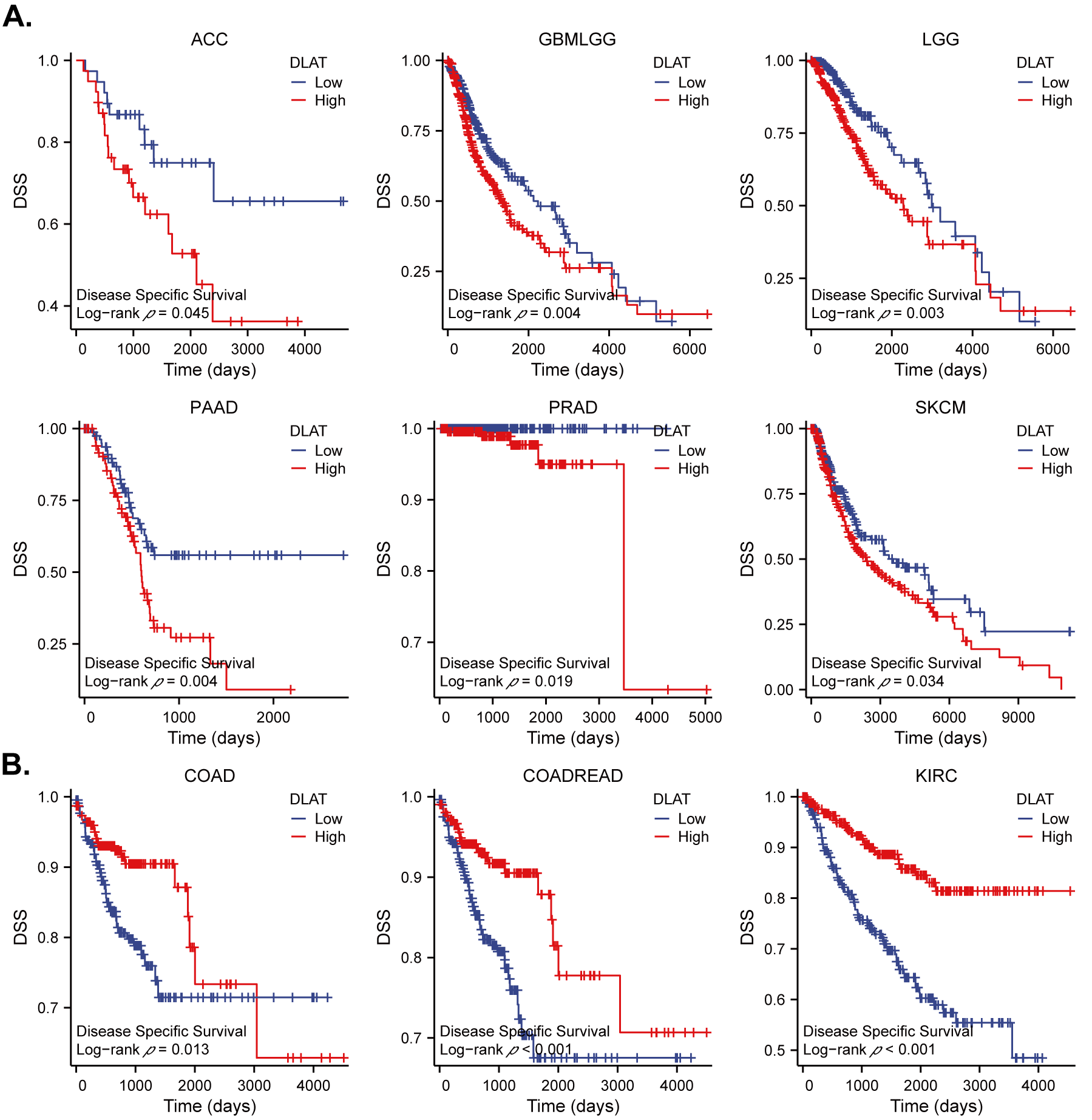

Supplement: Figure S4 [file OncolRes-32-48138-s004.tif]

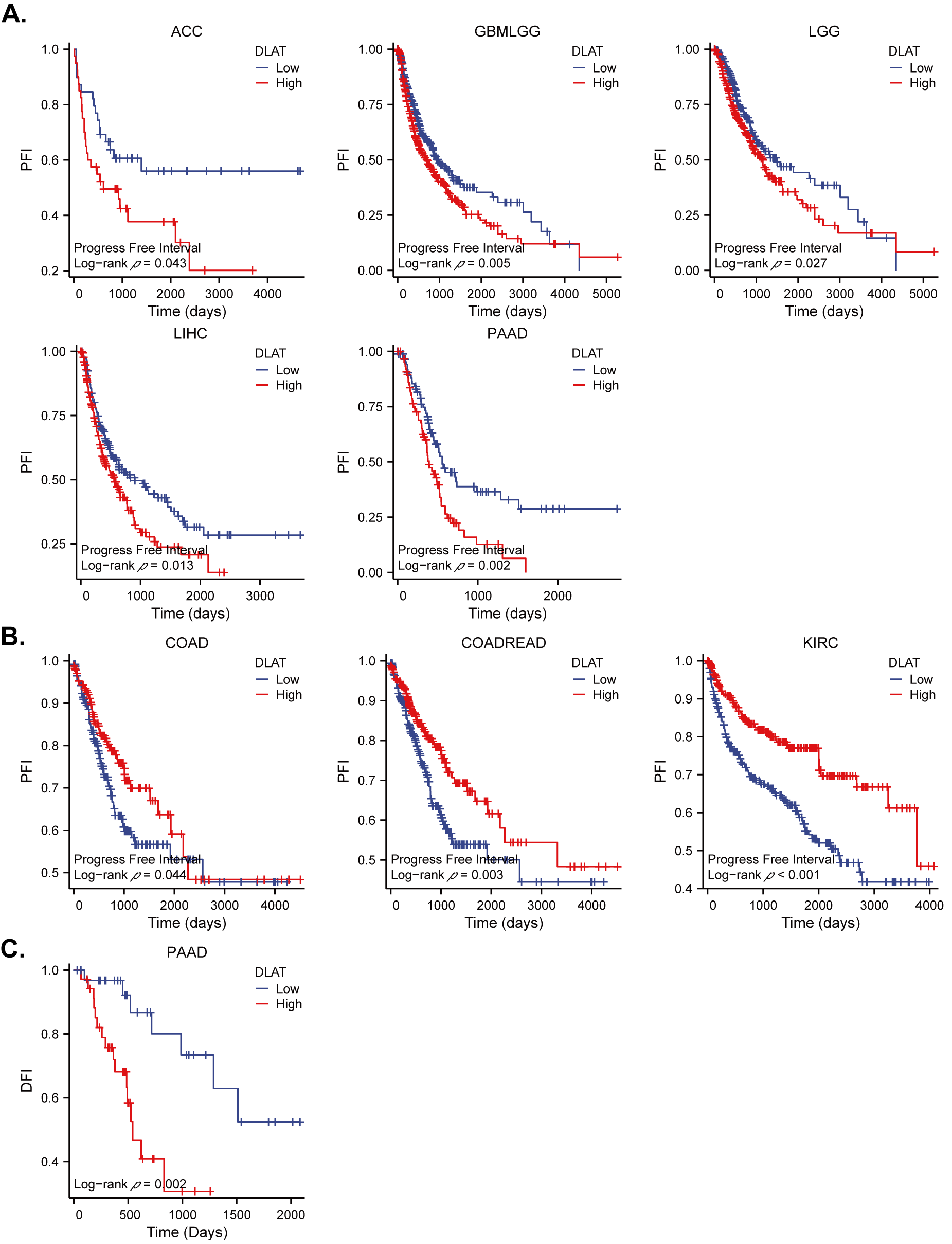

Supplement: Figure S5 [file OncolRes-32-48138-s005.tif]

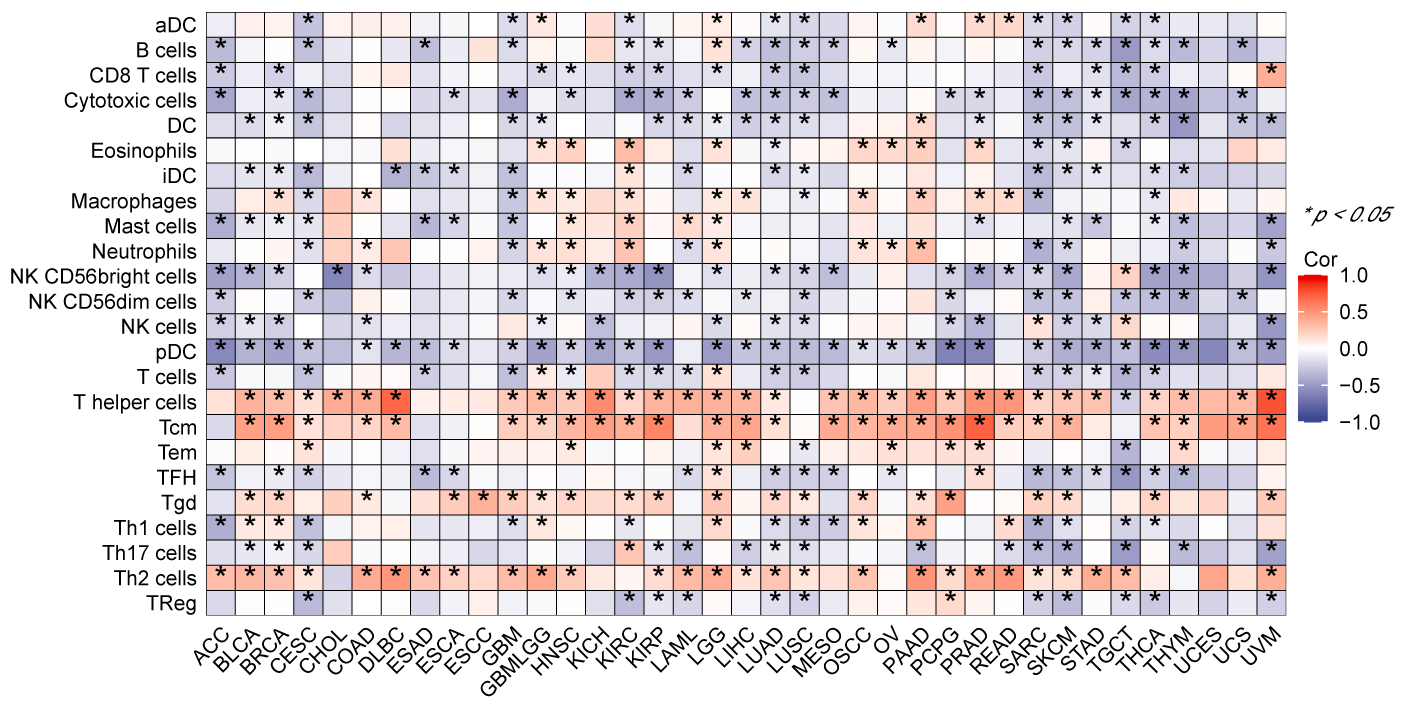

Supplement: Figure S6 [file OncolRes-32-48138-s006.tif]

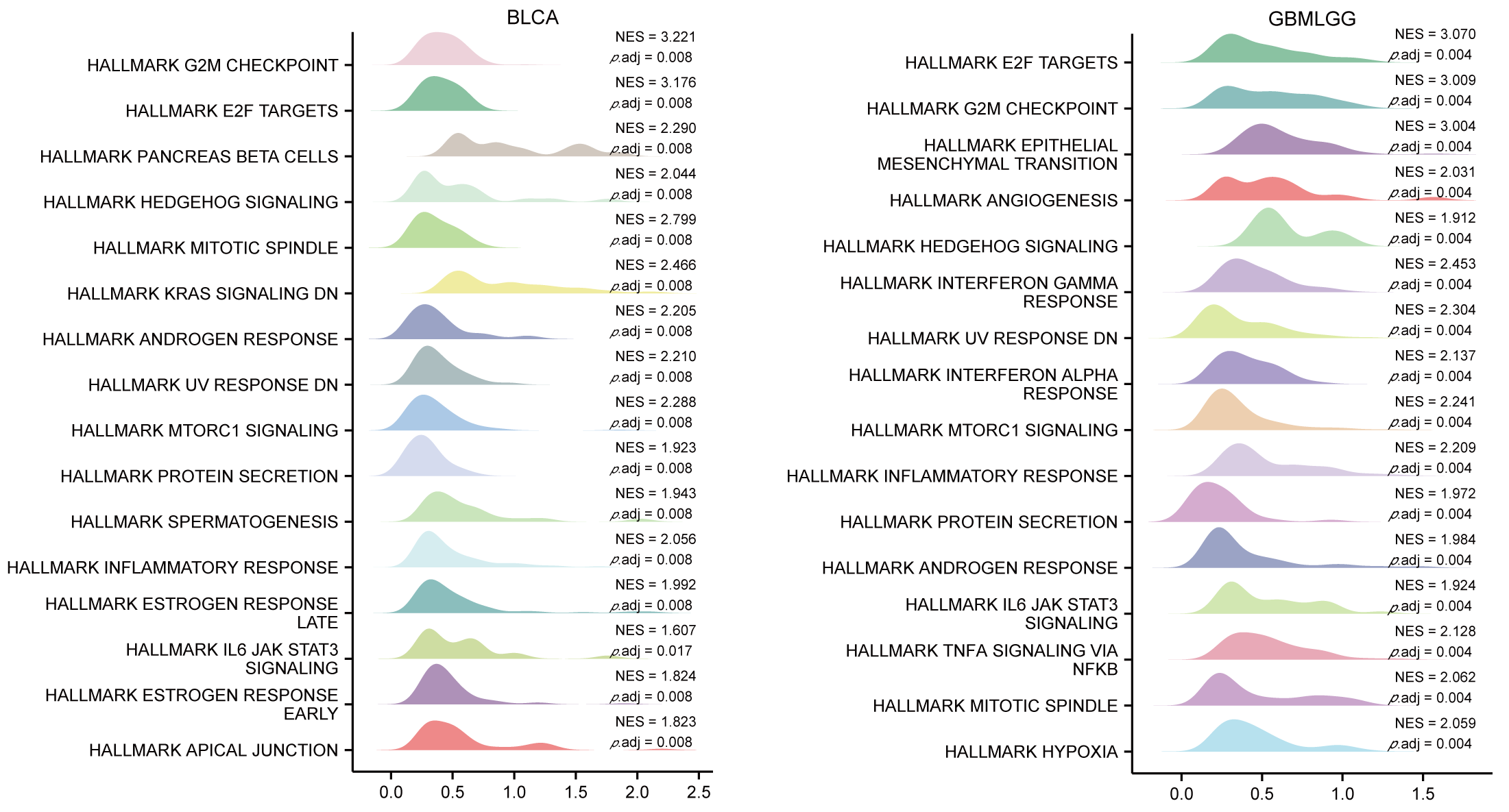

Supplement: Figure S7 [file OncolRes-32-48138-s007.tif]

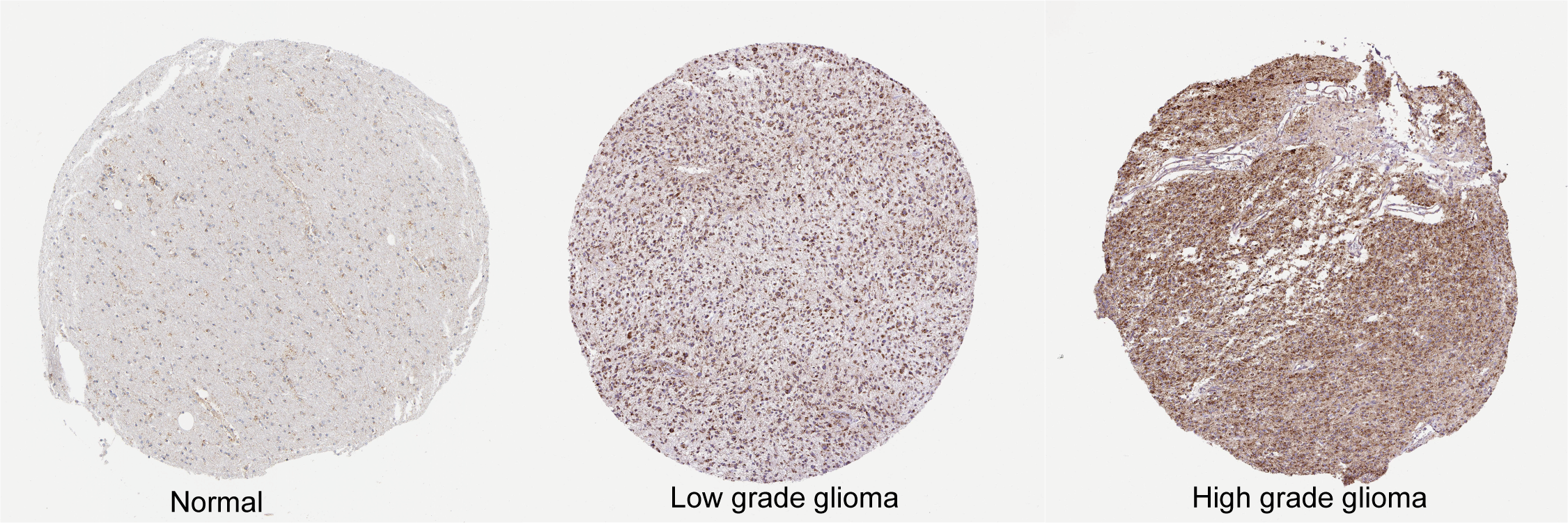

Supplement: Figure S8 [file OncolRes-32-48138-s008.tif]

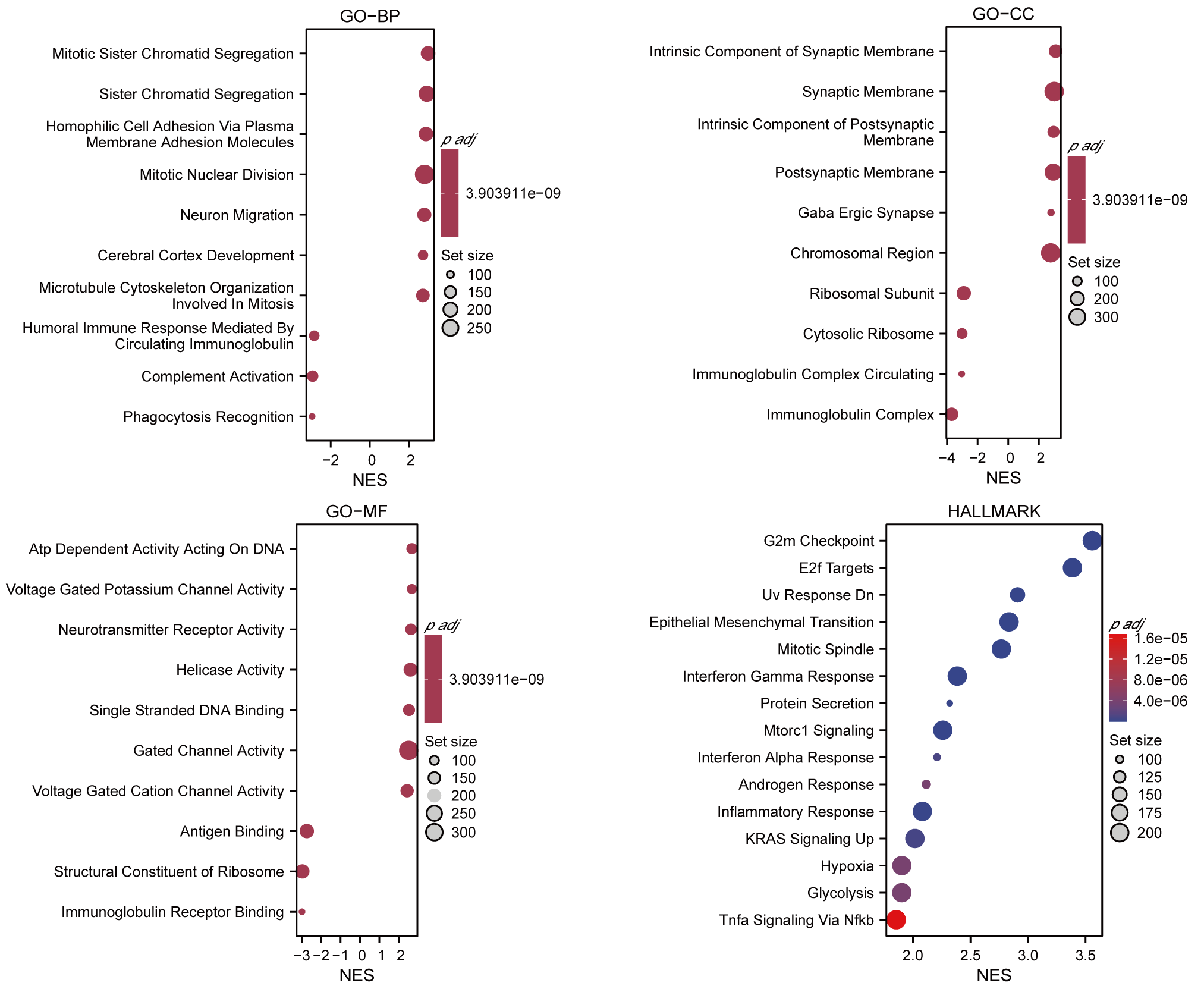

Supplement: Figure S9 [file OncolRes-32-48138-s009.tif]

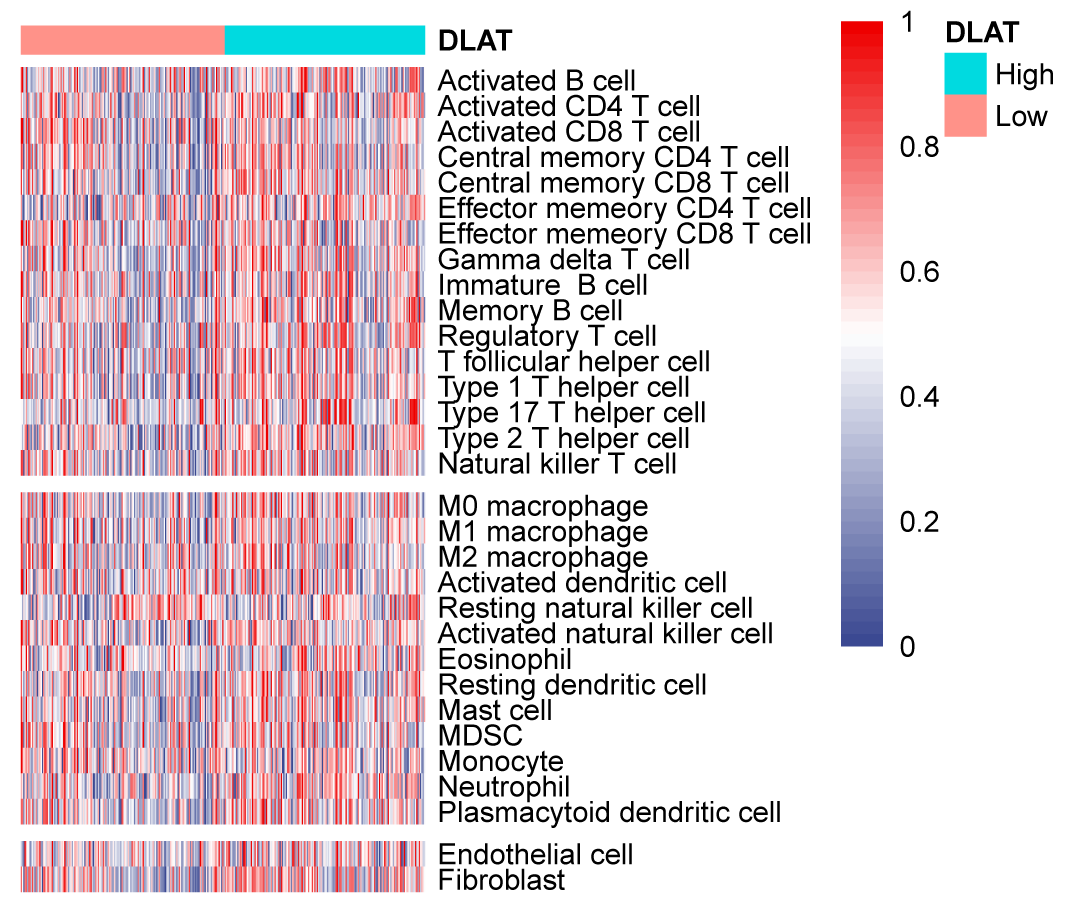

Supplement: Figure S10 [file OncolRes-32-48138-s010.tif]
