# Supplementary material for: A comprehensive and systematic analysis of Dihydrolipoamide S-acetyltransferase (DLAT) as a novel prognostic biomarker in pan-cancer and glioma
Source: Oncol Res. 2024 Nov 13;32(12):1903–19. doi: 10.32604/or.2024.048138 (PMC11576973; doi:10.32604/or.2024.048138)
Supplement: Supplementary file 11 [file OncolRes-32-48138-s001.docx]

| **Table S1. The full names and corresponding abbreviations of tumors.** | |
| --- | --- |
| Cohorts | Full names |
| TCGA-ACC | Adrenocortical carcinoma |
| TCGA-BLCA | Bladder Urothelial Carcinoma |
| TCGA-BRCA | Breast invasive carcinoma |
| TCGA-CESC | Cervical squamous cell carcinoma and endocervical adenocarcinoma |
| TCGA-CHOL | Cholangiocarcinoma |
| TCGA-COAD | Colon adenocarcinoma |
| TCGA-COADREAD | Colon adenocarcinoma/Rectum adenocarcinoma Esophageal carcinoma |
| TCGA-DLBC | Lymphoid Neoplasm Diffuse Large B-cell Lymphoma |
| TCGA-ESCA | Esophageal carcinoma |
| TCGA-FPPP | FFPE Pilot Phase II |
| TCGA-GBM | Glioblastoma multiforme |
| TCGA-GBMLGG | Glioma |
| TCGA-HNSC | Head and Neck squamous cell carcinoma |
| TCGA-KICH | Kidney Chromophobe |
| TCGA-KIPAN | Pan-kidney cohort (KICH+KIRC+KIRP) |
| TCGA-KIRC | Kidney renal clear cell carcinoma |
| TCGA-KIRP | Kidney renal papillary cell carcinoma |
| TCGA-LAML | Acute Myeloid Leukemia |
| TCGA-LGG | Brain Lower Grade Glioma |
| TCGA-LIHC | Liver hepatocellular carcinoma |
| TCGA-LUAD | Lung adenocarcinoma |
| TCGA-LUSC | Lung squamous cell carcinoma |
| TCGA-MESO | Mesothelioma |
| TCGA-OV | Ovarian serous cystadenocarcinoma |
| TCGA-PAAD | Pancreatic adenocarcinoma |
| TCGA-PCPG | Pheochromocytoma and Paraganglioma |
| TCGA-PRAD | Prostate adenocarcinoma |
| TCGA-READ | Rectum adenocarcinoma |
| TCGA-SARC | Sarcoma |
| TCGA-STAD | Stomach adenocarcinoma |
| TCGA-SKCM | Skin Cutaneous Melanoma |
| TCGA-STES | Stomach and Esophageal carcinoma |
| TCGA-TGCT | Testicular Germ Cell Tumors |
| TCGA-THCA | Thyroid carcinoma |
| TCGA-THYM | Thymoma |
| TCGA-UCEC | Uterine Corpus Endometrial Carcinoma |
| TCGA-UCS | Uterine Carcinosarcoma |
| TCGA-UVM | Uveal Melanoma |
| TARGET-OS | Osteosarcoma |
| TARGET-ALL | Acute Lymphoblastic Leukemia |
| TARGET-NB | Neuroblastoma |
| TARGET-WT | High-Risk Wilms Tumor |

**Table S2. Details of diagnostic ROC for *DLAT* in pan-cancer.**

| **Tumor type** | **Tumor(n)** | **Normal(n)** | **AUC(CI)** | **cut-off** | **sensitivity** | **specificity** | **Positive predictive value** | **Negative predictive value** | **YI** |
| --- | --- | --- | --- | --- | --- | --- | --- | --- | --- |
| **ACC** | **77** | **128** | **0.616 (0.530-0.702)** | **3.464** | **0.506** | **0.805** | **0.609** | **0.730** | **0.311** |
| **BLCA** | **407** | **28** | **0.615 (0.529-0.701)** | **3.918** | **0.607** | **0.714** | **0.969** | **0.111** | **0.321** |
| **BRCA** | **1099** | **292** | **0.577 (0.540-0.613)** | **3.913** | **0.702** | **0.452** | **0.828** | **0.287** | **0.154** |
| **CESC** | **306** | **13** | **0.580 (0.449-0.711)** | **4.102** | **0.291** | **1.000** | **1.000** | **0.057** | **0.291** |
| **CHOL** | **36** | **9** | **0.951 (0.852-1.000)** | **3.485** | **0.889** | **1.000** | **1.000** | **0.973** | **0.889** |
| **COAD** | **290** | **349** | **0.784 (0.748-0.819)** | **4.397** | **0.679** | **0.791** | **0.730** | **0.748** | **0.470** |
| **DLBC** | **47** | **444** | **0.796 (0.755-0.836)** | **2.061** | **1.000** | **0.707** | **0.266** | **1.000** | **0.707** |
| **ESAD** | **80** | **10** | **0.741 (0.605-0.877)** | **4.936** | **0.900** | **0.588** | **0.214** | **0.979** | **0.488** |
| **ESCA** | **182** | **666** | **0.809 (0.772-0.847)** | **4.410** | **0.654** | **0.832** | **0.515** | **0.898** | **0.486** |
| **GBM** | **166** | **1157** | **0.877 (0.850-0.904)** | **3.978** | **0.801** | **0.821** | **0.391** | **0.966** | **0.622** |
| **HNSC** | **502** | **44** | **0.685 (0.612-0.758)** | **4.800** | **0.795** | **0.596** | **0.147** | **0.971** | **0.391** |
| **KICH** | **66** | **53** | **0.707 (0.615-0.799)** | **5.260** | **0.500** | **0.868** | **0.825** | **0.582** | **0.368** |
| **KIRC** | **531** | **100** | **0.712 (0.645-0.780)** | **4.943** | **0.896** | **0.550** | **0.914** | **0.500** | **0.446** |
| **KIRP** | **289** | **60** | **0.552 (0.466-0.639)** | **4.768** | **0.758** | **0.433** | **0.866** | **0.271** | **0.191** |
| **LAML** | **173** | **70** | **1.000 (1.000-1.000)** | **5.542** | **1.000** | **1.000** | **1.000** | **1.000** | **1.000** |
| **LGG** | **523** | **1152** | **0.837 (0.817-0.857)** | **3.795** | **0.793** | **0.730** | **0.572** | **0.886** | **0.524** |
| **LIHC** | **371** | **160** | **0.723 (0.679-0.768)** | **2.872** | **0.679** | **0.694** | **0.837** | **0.483** | **0.373** |
| **LUAD** | **515** | **347** | **0.811 (0.783-0.839)** | **4.245** | **0.588** | **0.908** | **0.904** | **0.598** | **0.496** |
| **LUSC** | **498** | **338** | **0.796 (0.766-0.825)** | **4.243** | **0.526** | **0.917** | **0.903** | **0.568** | **0.443** |
| **OSCC** | **329** | **32** | **0.653 (0.558-0.748)** | **4.800** | **0.750** | **0.581** | **0.148** | **0.960** | **0.331** |
| **OV** | **427** | **88** | **0.875 (0.844-0.905)** | **3.606** | **0.754** | **0.909** | **0.976** | **0.432** | **0.663** |
| **PAAD** | **179** | **171** | **0.968 (0.948-0.988)** | **2.743** | **0.939** | **0.936** | **0.939** | **0.936** | **0.874** |
| **PRAD** | **496** | **152** | **0.623 (0.573-0.673)** | **3.593** | **0.633** | **0.618** | **0.844** | **0.341** | **0.251** |
| **READ** | **93** | **318** | **0.807 (0.748-0.866)** | **4.438** | **0.677** | **0.868** | **0.600** | **0.902** | **0.545** |
| **SKCM** | **469** | **813** | **0.577 (0.542-0.611)** | **3.984** | **0.529** | **0.625** | **0.453** | **0.702** | **0.164** |
| **STAD** | **414** | **210** | **0.890 (0.864-0.915)** | **3.798** | **0.821** | **0.810** | **0.895** | **0.697** | **0.631** |
| **TGCT** | **154** | **165** | **0.582 (0.516-0.648)** | **3.819** | **0.455** | **0.818** | **0.700** | **0.616** | **0.273** |
| **THCA** | **512** | **338** | **0.601 (0.563-0.639)** | **4.164** | **0.512** | **0.731** | **0.742** | **0.497** | **0.242** |
| **THYM** | **119** | **446** | **0.756 (0.716-0.795)** | **1.862** | **0.992** | **0.679** | **0.452** | **0.997** | **0.671** |
| **UCEC** | **181** | **101** | **0.611 (0.546-0.675)** | **4.032** | **0.387** | **0.960** | **0.946** | **0.466** | **0.347** |
| **UCS** | **57** | **78** | **0.624 (0.522-0.725)** | **4.026** | **0.333** | **0.962** | **0.864** | **0.664** | **0.295** |

| **Table S3. Summary of Spearman’s correlation between *DLAT* expression and drug response (IC50 value) in cancer cell lines based upon the GDSC dataset.** | | |
| --- | --- | --- |
| **Compound** | **Correlation** | **P** |
| **rTRAIL** | **-0.138285142** | **0.000393553** |
| **Belinostat** | **-0.136070132** | **0.000101574** |
| **XMD14-99** | **-0.12641635** | **0.000223927** |
| **TL-2-105** | **-0.125302163** | **0.000244454** |
| **Tubastatin A** | **-0.122621849** | **0.000332646** |
| **Selisistat** | **-0.120096717** | **0.000532837** |
| **SGC0946** | **-0.119497198** | **0.000675531** |
| **T0901317** | **-0.119028544** | **0.000471267** |
| **CAY10603** | **-0.117826556** | **0.000687557** |
| **Alectinib** | **-0.116891385** | **0.00083624** |
| **OSI-930** | **-0.115912064** | **0.000769421** |
| **AZD6482** | **0.114984277** | **0.003568771** |
| **GW-2580** | **-0.113730197** | **0.001333019** |
| **Zibotentan** | **-0.11367807** | **0.001001525** |
| **Phenformin** | **-0.111934586** | **0.001692347** |
| **Ruxolitinib** | **-0.111427362** | **0.001299199** |
| **PIK-93** | **-0.111069461** | **0.001377785** |
| **Tivozanib** | **-0.109847262** | **0.001518348** |
| **TAK-715** | **-0.109469185** | **0.001339334** |
| **Quizartinib** | **-0.109298822** | **0.001514599** |
| **XMD15-27** | **-0.108947761** | **0.001777113** |
| **KIN001-236** | **-0.107858055** | **0.001788633** |
| **XMD13-2** | **-0.106913317** | **0.001949522** |
| **BMS-345541** | **-0.106701129** | **0.00186654** |
| **WZ3105** | **-0.106655586** | **0.002212037** |
| **Refametinib** | **0.106602453** | **0.002454976** |
| **5-Fluorouracil** | **-0.105833006** | **0.002404151** |
| **Ispinesib Mesylate** | **-0.105601196** | **0.00253937** |
| **Methotrexate** | **-0.105308162** | **0.002368977** |
| **Sepantronium bromide** | **-0.104759928** | **0.028461795** |
| **GSK1904529A** | **-0.103636123** | **0.024827492** |
| **CP466722** | **-0.101822553** | **0.003733289** |
| **UNC1215** | **-0.101494357** | **0.003570742** |
| **BIX02189** | **-0.098247837** | **0.004916336** |
| **KIN001-270** | **-0.096439753** | **0.00546409** |
| **Midostaurin** | **0.096125469** | **0.023426266** |
| **AKT inhibitor VIII** | **-0.095098972** | **0.007613229** |
| **STF-62247** | **-0.093811317** | **0.006815099** |
| **CUDC-101** | **-0.093370402** | **0.008952327** |
| **TL-1-85** | **-0.093119575** | **0.007672618** |
| **GSK1070916** | **-0.091759585** | **0.008818782** |
| **Amuvatinib** | **-0.090261899** | **0.009388374** |
| **Fedratinib** | **-0.090223595** | **0.010911287** |
| **JW-7-24-1** | **-0.090079564** | **0.010723587** |
| **NG-25** | **-0.089582372** | **0.0108238** |
| **Trametinib** | **0.088445257** | **0.012320759** |
| **PFI-3** | **-0.088358397** | **0.012251454** |
| **Selumetinib** | **0.088011915** | **0.015599655** |
| **PI-103** | **-0.087650277** | **0.013414629** |
| **GSK690693** | **-0.087257224** | **0.013770204** |
| **KIN001-266** | **-0.086273895** | **0.017869128** |
| **UNC0638** | **-0.085123351** | **0.015949036** |
| **Enzastaurin** | **-0.084973785** | **0.027153678** |
| **ZSTK474** | **-0.084718102** | **0.01961697** |
| **CX-5461** | **-0.084696287** | **0.016513299** |
| **PD0325901** | **0.084285385** | **0.019267928** |
| **Vorinostat** | **-0.083212195** | **0.015882896** |
| **CP724714** | **-0.08318285** | **0.028022694** |
| **OSI-027** | **-0.083147602** | **0.018799727** |
| **Tanespimycin** | **0.08243496** | **0.019096024** |
| **Masitinib** | **-0.081806292** | **0.019781648** |
| **QS11** | **-0.080614576** | **0.034811856** |
| **SNX-2112** | **-0.079257241** | **0.026371738** |
| **Idelalisib** | **-0.077401733** | **0.032489321** |
| **VX-11e** | **-0.076411383** | **0.04159261** |
| **VX-702** | **-0.07623772** | **0.032746522** |
| **QL-XI-92** | **-0.07580014** | **0.031018515** |
| **Navitoclax** | **-0.075711852** | **0.037179099** |
| **AICA Ribonucleotide** | **-0.075668884** | **0.038700738** |
| **Y-39983** | **-0.075604118** | **0.032791392** |
